# Polymerization of recombinant tau core fragments *in vitro* and seeding studies in cultured cells

**DOI:** 10.3389/fnins.2023.1268360

**Published:** 2023-12-15

**Authors:** Giavanna Paterno, Brach M. Bell, Alexis Riley-DiPaolo, Matthew J. LaVoie, Benoit I. Giasson

**Affiliations:** ^1^Department of Neuroscience, College of Medicine, University of Florida, Gainesville, FL, United States; ^2^Center for Translational Research in Neurodegenerative Disease, College of Medicine, University of Florida, Gainesville, FL, United States; ^3^Department of Neurology, College of Medicine, University of Florida, Gainesville, FL, United States; ^4^McKnight Brain Institute, College of Medicine, University of Florida, Gainesville, FL, United States

**Keywords:** Alzheimer’s disease, core fragments, corticobasal degeneration, Pick’s disease, prion-like, seeding, tau, tauopathy

## Abstract

The relative polymerization of specific tau protein cores that define Alzheimer’s disease, Pick’s disease and corticobasal degeneration were investigated using amyloid fluorometry and electron microscopy. In addition, the relative prion-like activities of polymers comprised of these respective tau protein segments were investigated in a cell-based assay. It is demonstrated that the seeding activities of specific tau core fibrils are affected by the presence of pathogenic tau missense mutations and the microtubule binding domain composition of tau. The unique impact of tau phosphorylation on seeding propensity was also investigated by altering stretches of phospho-mimetic and phospho-null residues in the presence of Alzheimer’s disease tau core fibrils. These results have important mechanistic implications for mutation and isoform-specific driven pathogenesis.

## Introduction

Tau is a microtubule (MT) associated protein which is expressed in neurons of the central nervous system (CNS) (Mandelkow and Mandelkow, 2012) and is present as 6 isoforms through alternative splicing of exons 2, 3, and 10 resulting in proteins with either 0, 1 or 2 N-terminal repeats and 3 or 4 MT binding repeats (Goedert et al., 1988, 1989a,b). Insoluble filamentous tau inclusions are found in several neurodegenerative diseases collectively known as tauopathies. These diseases include Alzheimer’s disease (AD), Pick’s disease (PiD), and corticobasal degeneration (CBD) (Wang and Mandelkow, 2015) and each differ in the morphology of the tau aggregates and the composition of tau isoforms within the respective pathological inclusions. AD tau aggregates are composed of all 6 tau isoforms, those found in PiD are predominantly composed of isoforms consisting of 3-repeat (3R) tau, while aggregates found in CBD predominately consist of 4-repeat (4R) tau (Goedert et al., 1992; Buée and Delacourte, 1999).

The hypothesized mechanism in which tau accumulates in the brain during disease has been compared to that of the prion protein, in which tau is able to become seed competent and induce a conformational change in naïve tau (Goedert, 2015; Goedert et al., 2017; Wischik et al., 2018). This hypothesis is supported by Braak staging used to describe tau deposition in AD in which tau aggregates such as neurofibrillary tangles (NFTs), dystrophic neurites (DNs), and neuropil threads (NTs) are found in anatomically connected areas in the human brain (Braak and Braak, 1991; Braak et al., 2006). Additionally, *in vivo* mouse models have been utilized to recapitulate this phenomenon by injecting lysate from individuals with tauopathies, recombinant tau fibrils, or lysate from transgenic mice into host mice with different genotypes to investigate the anatomical distribution of tau aggregation (Clavaguera et al., 2013; Narasimhan et al., 2017; He et al., 2020; Weitzman et al., 2020; Xu et al., 2021).

Pathological hallmarks of AD are composed of paired helical filaments (PHFs) and straight filaments (SFs) (Goedert et al., 1992). Historically it has been observed that PHFs and SFs consist of a pronase resistant core composed of part of the MT binding domain (MTBD), while the N- and C- terminus of tau make up the fuzzy coat of the filaments, as visualized by electron microscopy (EM) (Wischik et al., 1988b). Structurally, these findings have been further explicated by cryo-EM studies which have elucidated the amino acid sequences of the filament core of both PHFs and SFs found in AD, as well as the tau cores of filaments found in PiD and CBD (Fitzpatrick et al., 2017; Falcon et al., 2018a,b; Zhang et al., 2020).

Full length tau and recombinant tau fragments known as K18 for 4-repeat tau and K19 for 3-repeat tau, are commonly incubated with heparin to induce tau polymerization (Goedert et al., 1996; Hasegawa et al., 1997; Iba et al., 2013; Strang et al., 2018; Xia et al., 2021). The latter truncated tau constructs lack several residues (373–380) that have been confirmed by cryo-EM to form the core of PHFs and SFs (Fitzpatrick et al., 2017; Falcon et al., 2018b). Tau fibrils assembled from the co-incubation of the MTBD of tau and polyanions such as heparin, have been widely used to study tau’s propensity to seed wild-type (WT) and mutant tau *in vitro* and *in vivo* (Guo and Lee, 2011; Iba et al., 2013; Chakrabarty et al., 2015; Strang et al., 2018; Xia et al., 2021), but heparin-induced tau fibrils do not completely recapitulate filaments found in the brains of patients with tauopathies (Zhang et al., 2019).

A previous report showed the tau core found in aggregates of several tauopathies, confirmed by cryo-EM, form filaments without being induced by heparin (Carlomagno et al., 2021). In our current study, we performed seeding experiments with 6 distinct tau fibrils based on the sequence of the cores found in the brains of individuals with sporadic or familial AD, PiD, and CBD. Our data demonstrate clear differences between the intrinsic propensity of WT 0N/3R and WT 0N/4R tau seeding-induced aggregation in cultured cells. Furthermore, 0N/4R tau with disease causal mutations display seeding activity similar to WT 0N/3R tau. These studies taken together with prior work assessing the effects of tau isoforms and mutants on tau-MT binding (Hong et al., 1998; Xia et al., 2019, 2021) suggest that tau’s ability to be seeded is highly dependent on the propensity of tau to dissociate from MTs.

## Materials and methods

### Key reagents

HEK293T cells were purchased from ATCC. Dulbecco’s Modified Eagle Medium, fetal bovine serum (FBS), and penicillin–streptomycin were purchased from Fisher Scientific (Hampton, NH). K114 was prepared previously (Crystal et al., 2003). Thioflavin T was purchased from MilliporeSigma (Burlington, MA).

### Generation and purification of tau amyloid core fragments

**Figure 1:**
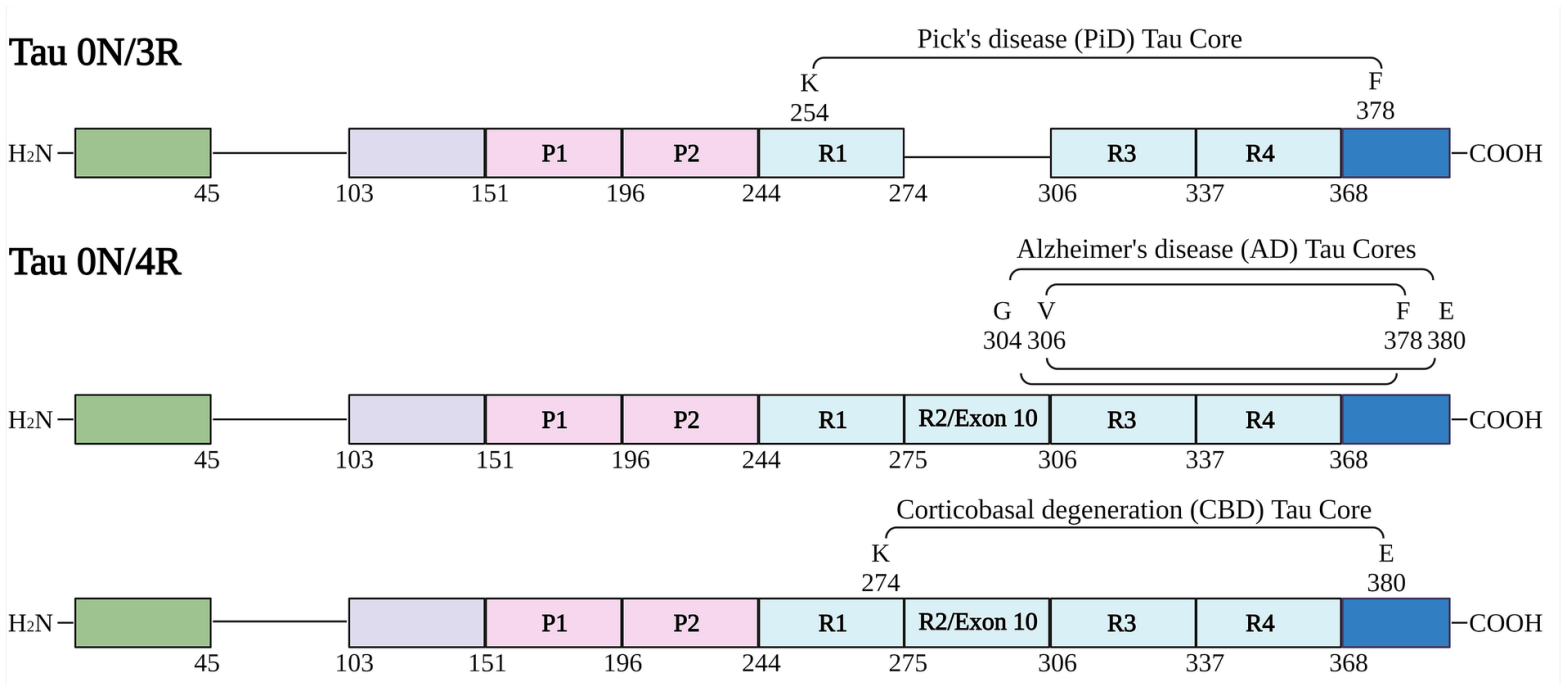
Schematic of human tau isoforms and fragments used in the study. Numbering is according to the longest human tau isoform in the central nervous system (2N/4R). Schematic shows tau fragments used in this study termed “cores” (based on prior cryo-EM structure studies of tau fibrils). Created with BioRender.com.

The cDNA encoding the various human tau fragments (see Figure 1) were amplified by PCR from plasmids with either 0N/3R or 0N/4R human tau cDNA and DNA primers with added 5’ *NdeI* restriction site (which adds an N-terminal ATG methionine codon for expression initiation) and C-terminal stop codon (TAA) and 3’ *EcoRI* restriction site. The PCR DNA fragments were purified, TA cloned, and subcloned into the bacterial expression vector pRK172 plasmid (Goedert and Jakes, 1990). Sanger sequencing of all tau fragments was performed by Genewiz/Azenta (South Plainfield, NJ).

The pRK172 bacterial expression vector containing the cDNAs for the various tau fragments were transformed into BL21 (DE3) *Escherichia coli* (*E. coli*; New England BioLabs Inc). The bacteria were grown in terrific broth with 100 μg/ml ampicillin to an optical density of 0.6 at 37°C and 0.5 mM isopropyl β-D-1-thiogalactopyranoside was added for 2 h. Cells were pelleted and resuspended by homogenization in high salt RAB buffer (750 mM NaCl, 20 mM NaF, 100 mM MES, pH 7.0, 1 mM EGTA, 0.5 mM MgSO_4_) with a cocktail of protease inhibitor (1 mM phenylmethylsulphonyl fluoride, 1 μg/mL each of pepstatin, leupeptin, *N*-tosyl-l-phenylalanyl chloromethyl ketone, *N*-tosyl-lysine chloromethyl ketone, and soybean trypsin inhibitor). The cells were lysed by heating to 100°C for 10 min. Cell debris were removed by centrifugation at 7000× *g* for 20 min. Supernatants were filtered through a 0.22-micron Steriflip filtration units (Millipore) and dialyzed overnight at 4°C against buffer A (20 mM PIPES, pH6.5, 10 mM NaCl, 1 mM EGTA, 1 mM MgSO_4_, 0.1 mM PMSF) and purified by cation exchanged chromatography with a HiTrap SP HP column (Cytiva) in buffer A with a 10–500 mM NaCl gradient. Protein concentrations were determine using bicinchoninic acid assay and bovine serum albumin as the standard.

### Preparation of tau core fibrils

Protein of various tau core fragments were diluted to 1 mg/mL in sterile phosphate buffered saline (PBS) and incubated with continuous shaking at 1050 RPM (Eppendorf Thermomixer C) at 37°C. For cell seeding, fibrils were sonicated for 1 h in a water bath sonicator (Branson CPX2800H; 40 kHz). For experiments using tau cores incubated with heparin, fibrils were prepared as above but with the addition of heparin at a final concentration of 50 μM.

### K114 and thioflavin T fluorometry

Both K114 and thioflavin T (ThT) are amyloid binding dyes with known excitation and emission spectra shifts allowing for the measuring of amyloid formation for proteins such as tau, α-synuclein, and amyloid-β peptides (LeVine III, 1993; Crystal et al., 2003). K114 is a derivative of Congo Red (Crystal et al., 2003). K114 and ThT have distinct amyloid binding sites (Levine, 2005). The K114 fluorescent assay was performed as previously described (Crystal et al., 2003) with 100 μM K114 in 100 mM glycine buffer, pH 8.6 and measuring fluorescence (λ_ex_ = 380 nm, λ_em_ = 550 nm, cutoff = 530 nm) using a Spectra-Max Gemini fluorometer and SoftMax Pro 4.0 software. ThT fluorometry was performed using ThT (20 μM) diluted into 100 mM glycine, pH 8.0 using the follow parameters: λ _ex_ = 450 nm, λ_em_ = 482 nm, cutoff = 475 nm. λ _ex_ and λ_em_ are parameters chosen from previous experiments performed with other amyloid proteins with cutoff optimized to reduce bleed-through from excitation to emission signal (LeVine III, 1993). Time point (*t* = 0, 24, 48, 72, 96 h) measurements were determined by adding the protein samples to K114 and ThT assay solutions. For each assay, 5 μL of protein (1 mg/mL) were added to 100 μL of K114 or ThT solutions.

### Cloning of phosphomimetic and null constructs

Constructs designated 12E/12A were mutated at the codon positions for serine (S) and threonine (T) residues in the proline rich region at residues S198, S199, S202, T205, S210, T212, S214, T217, T231, S235, S237, S238 to a glutamate (GAA codon) or alanine (GCC codon), respectively. Constructs designated 9E/9A were mutated at the codon positions for serine (S), threonine (T), and tyrosine (Y) sites in the C terminus at residues Y394, S396, S400, T403, S404, S409, S412, S422, S435 to a glutamate (GAA codon) or alanine (GCC codon) respectively. In some constructs, the codon for residue Pro301 was mutated to leucine (CTG codon) or residue R406 was mutated to tryptophan (TGG). Mutations were made in pcDNA3.1-0N/4R human tau and pcDNA3.1-0N/3R human tau. The constructs were generated as a service by GenScript (Piscataway, NJ) and confirmed by Sanger sequencing.

### Cell culture and calcium phosphate transfection

HEK293T cells were cultured at 37°C and 5% CO_2_ in Dulbecco’s modified Eagle’s media and 10% FBS with added antibiotics (100 units/mL penicillin, 100 μg/mL streptomycin). Different plasmids expressing human tau were transfected into HEK293T cells by calcium phosphate precipitation (Xia et al., 2019). Cells were plated into 12-well plates at 20–40% confluency. For each well, 1.5 μg of DNA was mixed with 18.75 μL of 0.25 M CaCl_2_. An equal amount of 2X BES buffer (50 mM BES, 280 mM NaCl, 1.5 mM Na_2_HPO_4_, pH 6.96) was added to this mixture and incubated for 15–20 min at room temperature. The final solution was added dropwise to each well. For tau seeding experiments, 8 μg of tau fibrils were added to wells containing 1 mL of media 1 h after transfection. 16 h after transfection, media was replaced and cells were grown in 3% FBS. Cells were harvested 48 h thereafter.

### Cell-based tau aggregation assay

HEK293T cells were lysed in 200 μL of Triton Lysis Buffer (25 mM Tris–HCl, pH 7.5, 150 mM NaCl, 1 mM EDTA, 1% Triton X-100, 20 mM NaF) with a mix of different protease inhibitors (see above). Cell lysates were centrifuged at 100,000× *g* at 4°C for 30 min to separate into soluble and insoluble fractions. The Triton soluble supernatant was kept and 200 μL of Triton Lysis Buffer was added to the pellet and centrifuged at 100,000× *g* at 4°C for 30 min. The wash supernatant was discarded and 200 μL of Triton Lysis Buffer was added to the pellet. SDS sample buffer (10 mM Tris, pH 6.8, 1 mM EDTA, 40 mM DTT, 0.005% bromophenol blue, 0.0025% pyronin yellow, 1% SDS, 10% sucrose) was added to the samples which were heated at 95°C for 10 min. The insoluble fraction was sonicated and heated again for 10 min to completely dissolve the pellet.

### Western blotting analysis

Equal volumes of each sample were loaded on 10% polyacrylamide gels and resolved by electrophoresis. After transfer to nitrocellulose membranes, the samples were blocked in 5% milk in Tris-buffered saline (TBS; 50 mM Tris, 150 mM NaCl, pH 7.5) for an hour. Membranes were incubated overnight at 4°C with primary antibodies 3026, 7F2, or PHF22 diluted 1:1000 in blocking solution (Strang et al., 2017) followed by goat anti-rabbit or goat anti-mouse secondary antibodies conjugated to horseradish peroxidase (Jackson Immuno Research labs, Westgrove, PA). After TBS washes, the membranes were exposed and imaged after adding Western Lightning Plus ECL reagents (PerkinElmer, Waltham, MA) followed by chemiluminescence imaging (PXi, Syngene, Frederick, MD). Tau signal in the immunoblots was measured using Image J software. A box was drawn that encompassed the major tau immuno-bands in the first lane and identical boxes were duplicated horizontally for all the other lanes within each immunoblot. Statistical analysis was performed using GraphPad Prism software (San Diego, CA). Two-way ANOVA multiple comparisons test with Tukey or Šidák correction for multiple comparisons was performed for western blot analysis. Reported *p* values are multiplicity adjusted.

### Antibodies

3026, 7F2, and PHF22 are anti-tau antibodies that have been described previously (Strang et al., 2017). 3026 is a total tau rabbit polyclonal antibody raised against 0N/3R human tau. 7F2 is a mouse monoclonal antibody that is specific for tau phosphorylated at Thr205. PHF22 is a mouse monoclonal antibody that is specific for tau phosphorylated at Ser396 and Ser404. Anti-actin clone C4 is a mouse monoclonal antibody (MilliporeSigma, Burlington, MA).

### Electron microscopy

Negative staining electron microscopy was performed on tau fibrils. 2 μL of tau fibrils (1 mg/mL) were applied to 300 mesh carbon coated copper grids (Electron Microscopy Sciences, Hatfield, PA) and were allowed to settle for 10 min. Grids were washed with filtered PBS. 1% uranyl acetate was filtered through a 2-micron filter and applied to each grid that were then dried. Samples were examined with a FEI Tecnai G2 Spirit Twin TEM (FEI Corp., Hillsboro, OR) operated at 120 kV and digital images were acquired with a Gatan UltraScan 2 k × 2 k camera and Digital Micrograph software (Gatan Inc., Pleasanton, CA).

## Results

### Tau cores readily fibrillize forming K114 and Thioflavin-T reactive amyloid structure

**Figure 2:**
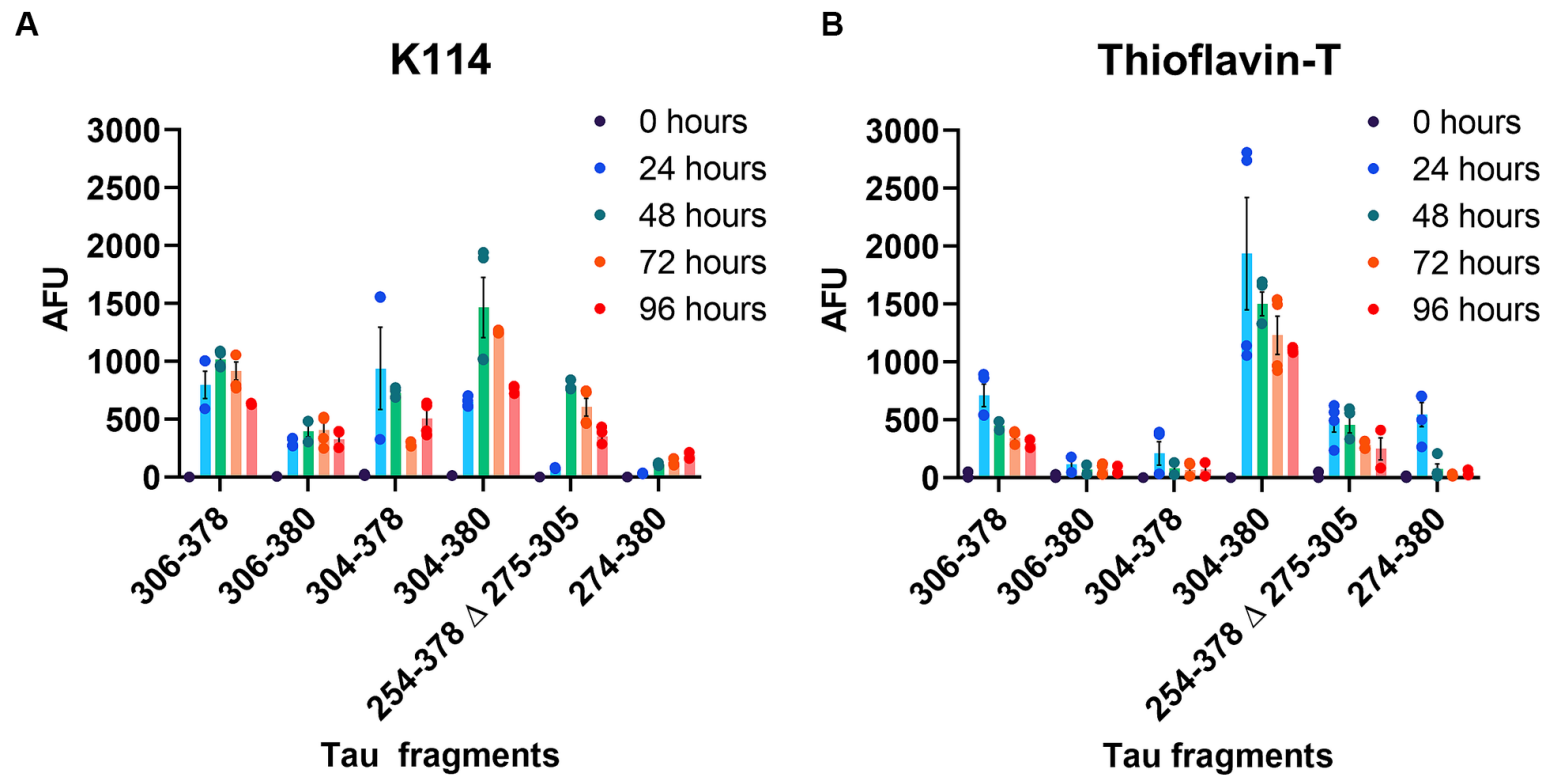
Comparative amyloid formation assessed by Thioflavin-T and K114 fluorometry. **(A)** K114 and **(B)** ThT dyes were used to monitor amyloid formation of each tau fragment at 0, 24, 48, 72, and 96 h as indicated with continuous shaking as described in “Materials and Methods”. *N* = 4. AFU, arbitrary fluorescence units. Data are shown as mean ± SEM.

Tau protein fragments corresponding to the amino acid sequence of AD, PiD and CBD core filament structures (Figure 1) were expressed and purified from bacteria as shown by Coomassie stained SDS-PAGE (Supplementary Figure S1). We further assessed amyloid formation with 2 independent amyloid binding dyes K114 and Thioflavin-T (ThT) (Friedhoff et al., 1998; Crystal et al., 2003). Amyloid formation was determined for each tau core at 0, 24, 48, 72, and 96 h. K114 amyloid measurements (Figure 2A) rapidly increased for 48 h for several tau cores (AD tau cores 306–378, 304–380, and variations 304–378 and 306–380 as well as PiD tau core 254–378 Δ 275–305) and plateaued or decreased thereafter. This is likely due to a conformational change in the amyloid binding pocket which disrupted the amyloid dye’s ability to bind to the tau fibrils. While these tau cores showed appreciable K114 fluorescence signal, the CBD tau core (274–380) showed comparably less robust amyloid formation based on K114.

**Figure 3:**
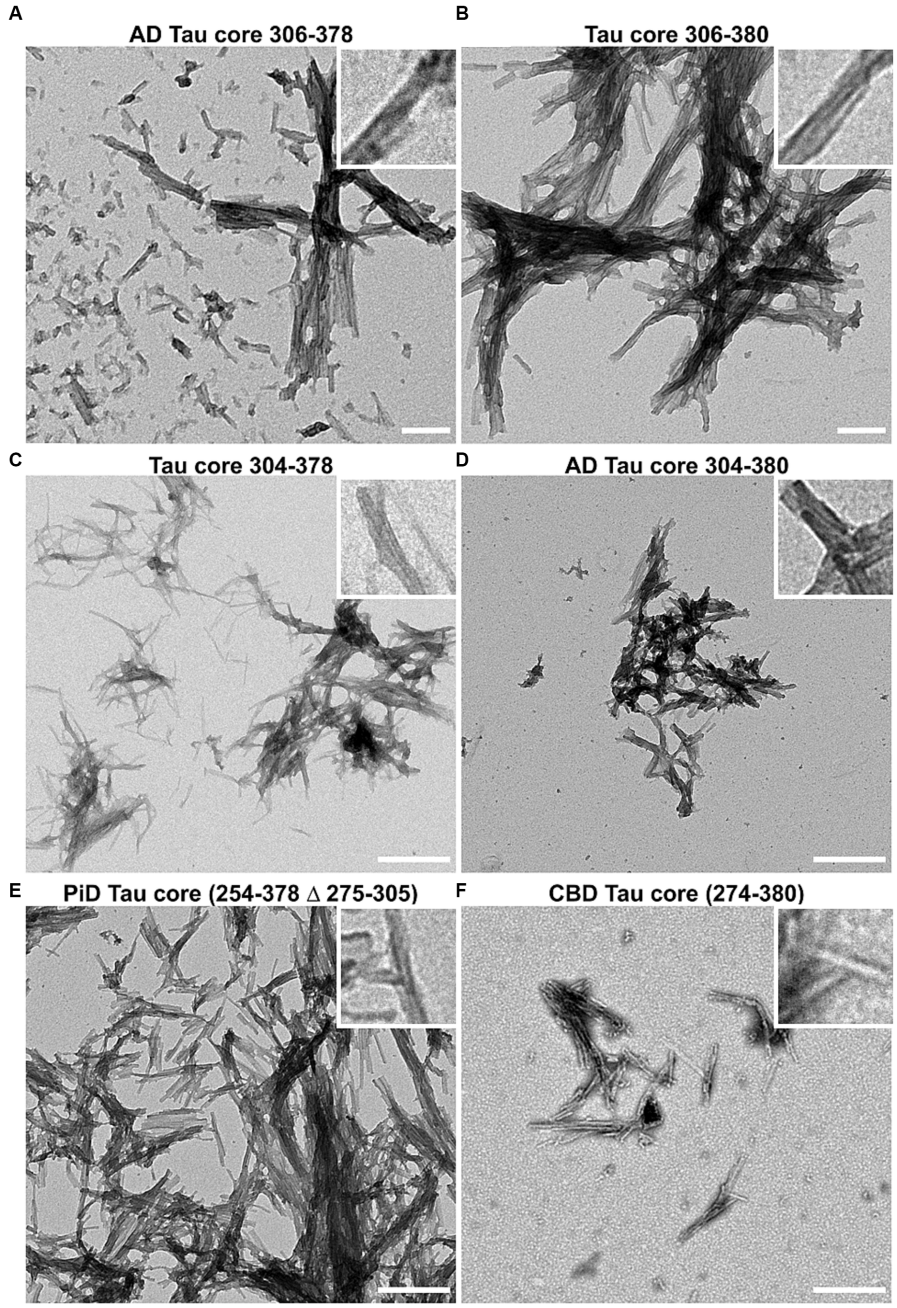
Negative staining electron microscopy of Tau core fibrils assembled *in vitro*. 6 tau cores were fibrillized and negatively stained with uranyl acetate and visualized by EM as described in “Materials and Methods”. Magnification for panels **(A,B)** was 14,753x and the scale bar is 200 nm (for insets 50 nm). Magnification for panels **(C–F)** is 8,838x and the scale bar is 500 nm (for insets 125 nm).

Amyloid formation measured by ThT displayed a similar trend (Figure 2B). Amyloid measurements of AD tau cores 306–378, 304–380, PiD tau core (254–378 Δ 275–305) and CBD tau core (274–380) increased and peaked by 24 h and then declined. By comparison, we observed that 2 of the 6 tau cores (tau cores 306–380, 304–378) exhibited limited amyloid formation by ThT. Tau core 304–380 exhibited the highest propensity for amyloid formation with both K114 and ThT (Figure 2). While K114 and ThT showed variable amyloid formation, the presence of fibril formation was further investigated by electron microscopy. Following *in vitro* incubation, samples were assessed by uranyl acetate staining and the presence of fibrils was confirmed for all 6 tau cores by electron microscopy (Figure 3). We observed predominantly straight fibrils without obvious twists, which varied in length depending on the field of view (Figure 3).

Charged polymers such as heparin are commonly used to induce the polymerization of full length and truncated tau proteins that do not readily aggregate by themselves (Goedert et al., 1996; Pérez et al., 1996; Friedhoff et al., 1998). We compared amyloid formation of AD tau core 306–378 in the presence and absence of heparin by K114 and ThT (Supplementary Figures S2A,B). By K114, amyloid formation in the presence and absence of heparin was similar. By ThT, amyloid formation of tau core 306–378 was higher in the absence of heparin, further indicating that inducers like heparin are not necessary to induce formation of fibrils composed of these amino acids of tau.

### Tau cores have variable seeding activity in a cell-based assay

**Figure 4:**
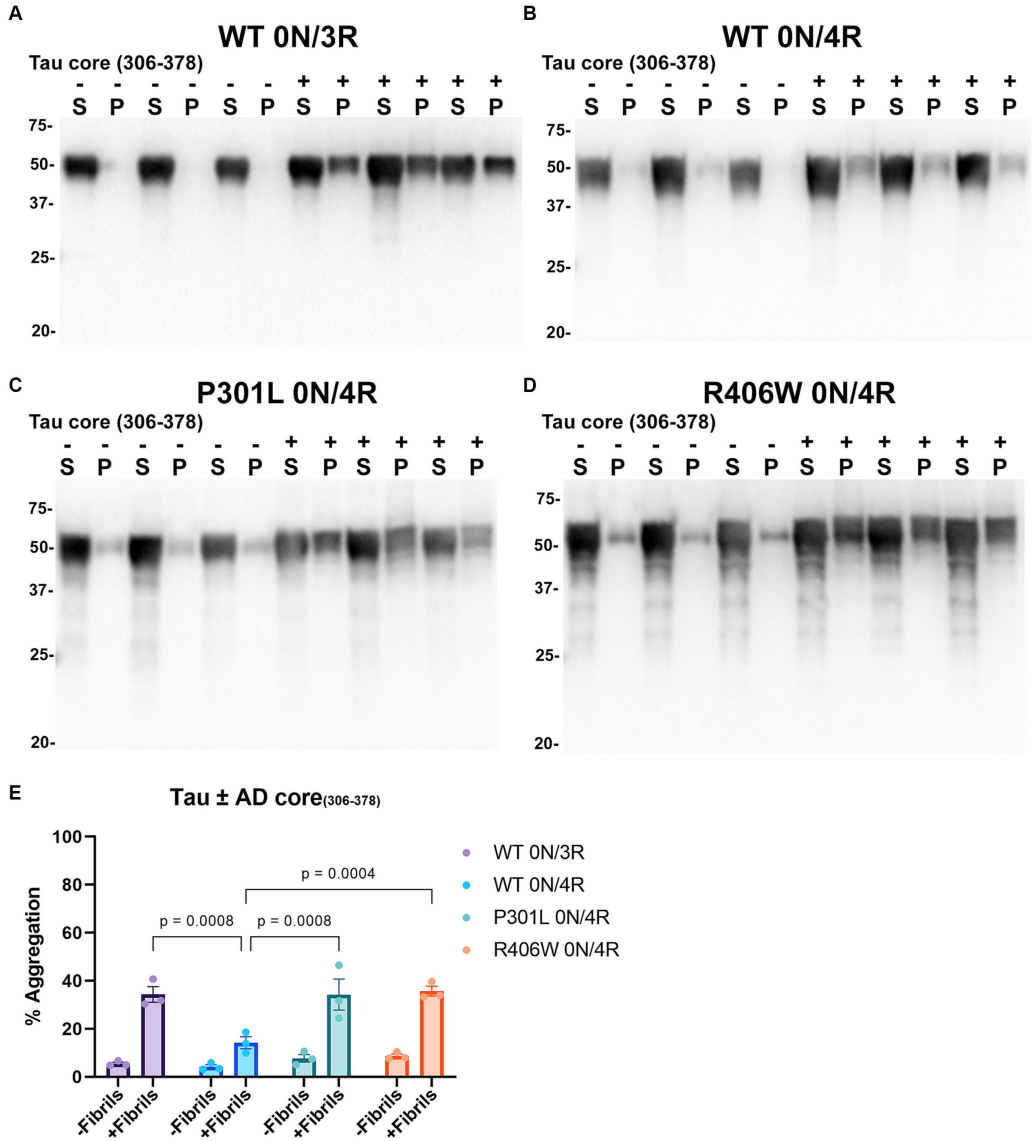
Immunoblot analyses of WT and Tau mutants seeded with the 306–378 AD tau core fibrils in HEK293T cells. Western blots of lysate from HEK293T cells transfected to express **(A)** 0N/3R, **(B)** 0N/4R, **(C)** P301L 0N/4R, or **(D)** R406W 0N/4R human tau. Cells were untreated (−) or treated (+) with fibrils assembled from the tau fragment corresponding to amino acids 306–378 numbered according to full length 2 N/4R tau. Blots were probed with rabbit polyclonal 3026. ‘S’ indicates Triton soluble factions and ‘P’ indicates Triton insoluble pellet fractions as indicated above each lane. The apparent mobilities of molecular weight markers are indicated on the left. **(E)** Percent aggregation was calculated using the formula [pellet/(pellet + supernatant)] × 100. *N* = 3 for each condition. Data are shown as the mean ± SEM. Two-way ANOVA was performed with correction for multiple comparisons using Tukey test. Statistically significant *p* values (*p* < 0.05) are indicated.

**Figure 5:**
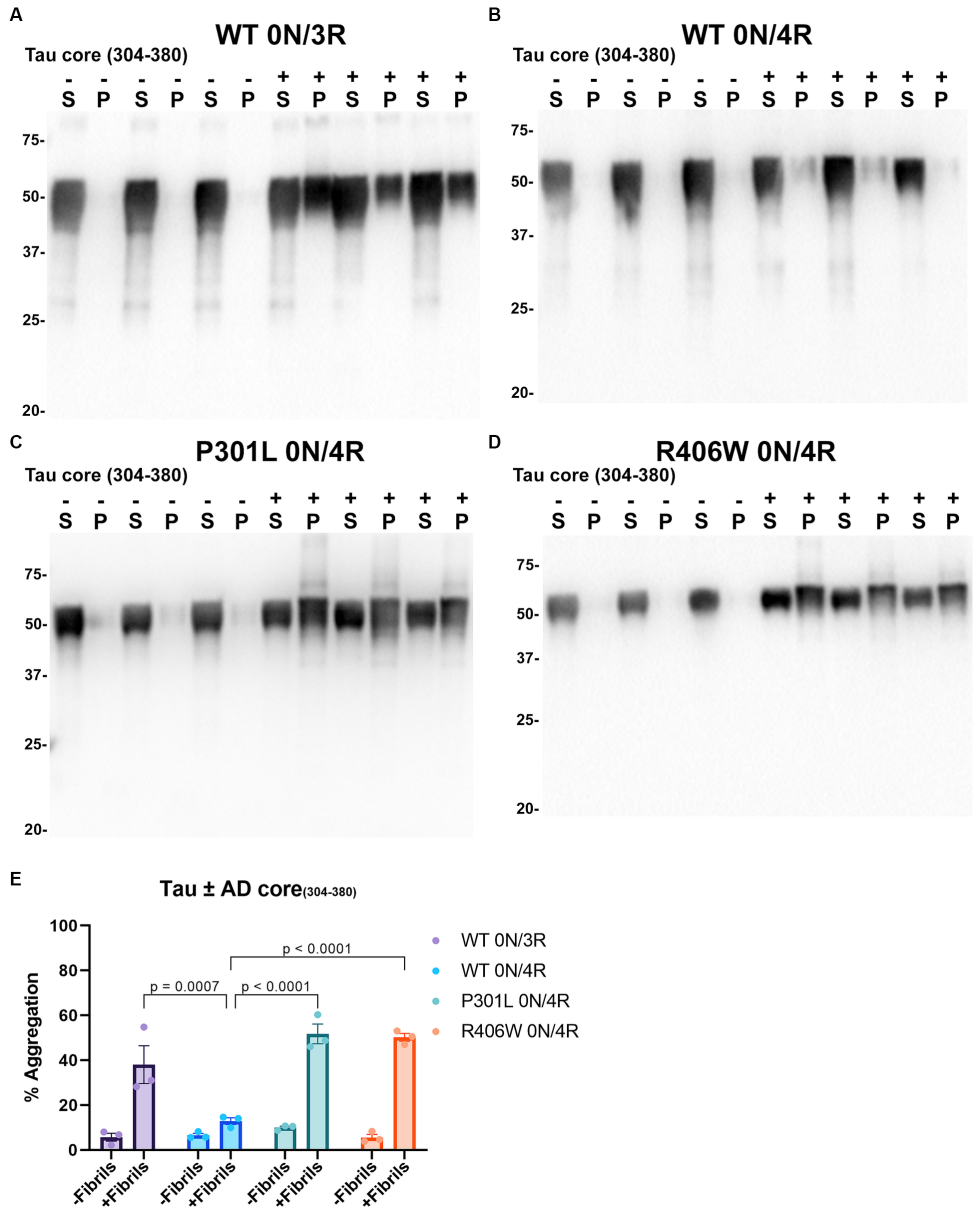
Immunoblot analyses of WT and mutant Tau mutants seeded with the 304–380 AD tau core fibrils in HEK293T cells. Western blots of lysate from HEK293T cells transfected to express **(A)** 0N/3R, **(B)** 0N/4R, **(C)** P301L 0N/4R, or **(D)** R406W 0N/4R human tau. Cells were untreated (−) or treated (+) with fibrils assembled from the tau fragments corresponding to amino acids 304–380 of full length 2 N/4R tau. Blots were probed with rabbit polyclonal 3026. ‘S’ indicates Triton soluble fractions and ‘P’ indicates Triton insoluble pellet fractions as indicated above each lane. The apparent mobilities of molecular weight markers are indicated on the left. **(E)** Percent aggregation was calculated using the formula [pellet/(pellet + supernatant)] × 100. *N* = 3 for each condition. Data are shown as the mean ± SEM. Two-way ANOVA was performed with correction for multiple comparisons using Tukey test. Statistically significant *p* values (*p* < 0.05) are indicated.

Tau cores used in this study were identified by cryo-EM as the core elements that comprise the pathological inclusions present in the brains of individuals with tauopathies such as AD, PiD, and CBD (Fitzpatrick et al., 2017; Falcon et al., 2018a,b; Zhang et al., 2020). Tau cores 304–380 and 306–378 were specifically identified from the brains of patients with AD, while tau cores 304–378 and 306–380 are altered versions of these. Tau core 254–378 Δ 275–305 was specifically associated with PiD filaments, and tau core 274–380 was present in CBD filaments. We conducted cell-based aggregation assays using these 6 distinct tau cores in the presence of over-expressed WT 0N/3R and 0N/4R tau isoforms as well as pathological mutants P301L 0N/4R and R406W 0N/4R for 3 of the tau cores, which in previous studies were shown to decrease MT interaction (Hong et al., 1998; Xia et al., 2019, 2021). Additionally, our group as well as others has investigated the pathogenic P301L mutation and its susceptibility for seeding and have previously established a seeding model using exogenous K18 fibrils in a mammalian cell culture system as it is more prone to aggregate with K18 tau seeds (Strang et al., 2018; Xia et al., 2019). Treatment of HEK293T cells expressing WT 0N/3R, P301L or R406W 0N/4R tau with exogenous AD tau core 306–378 polymers resulted in Triton X-100 (Triton) insoluble tau aggregates and more so than cells expressing WT 0N/4R tau (Figure 4). We also investigated if seeding propensity would change when tau core 306–378 was fibrillized in the presence of heparin (Supplementary Figure S2). We observed an increase in Triton insoluble tau material when isoforms 0N/3R and 0N/4R and mutants P301L and R406W 0N/4R tau were seeded with AD tau core 306–378 (Supplementary Figures S2C–F). These findings were similar to seeding with tau core 306–378 fibrillized without heparin (Figure 4), except that 0N/4R tau could also be readily seeded with the heparin-treated AD tau core 306–378 polymers. Moreover, there was a difference observed where WT and P301L 0N/4R tau accumulated more Triton insoluble aggregates compared to R406W 0N/4R tau (Supplementary Figure S2G). AD tau core 304–380 seeds were also potent at seeding WT 0N/3R, P301L 0N/4R, and R406W 0N/4R tau while the induced aggregation for WT 0N/4R tau was much lower (Figure 5), similar to the findings with AD tau core 306–378.

**Figure 6:**
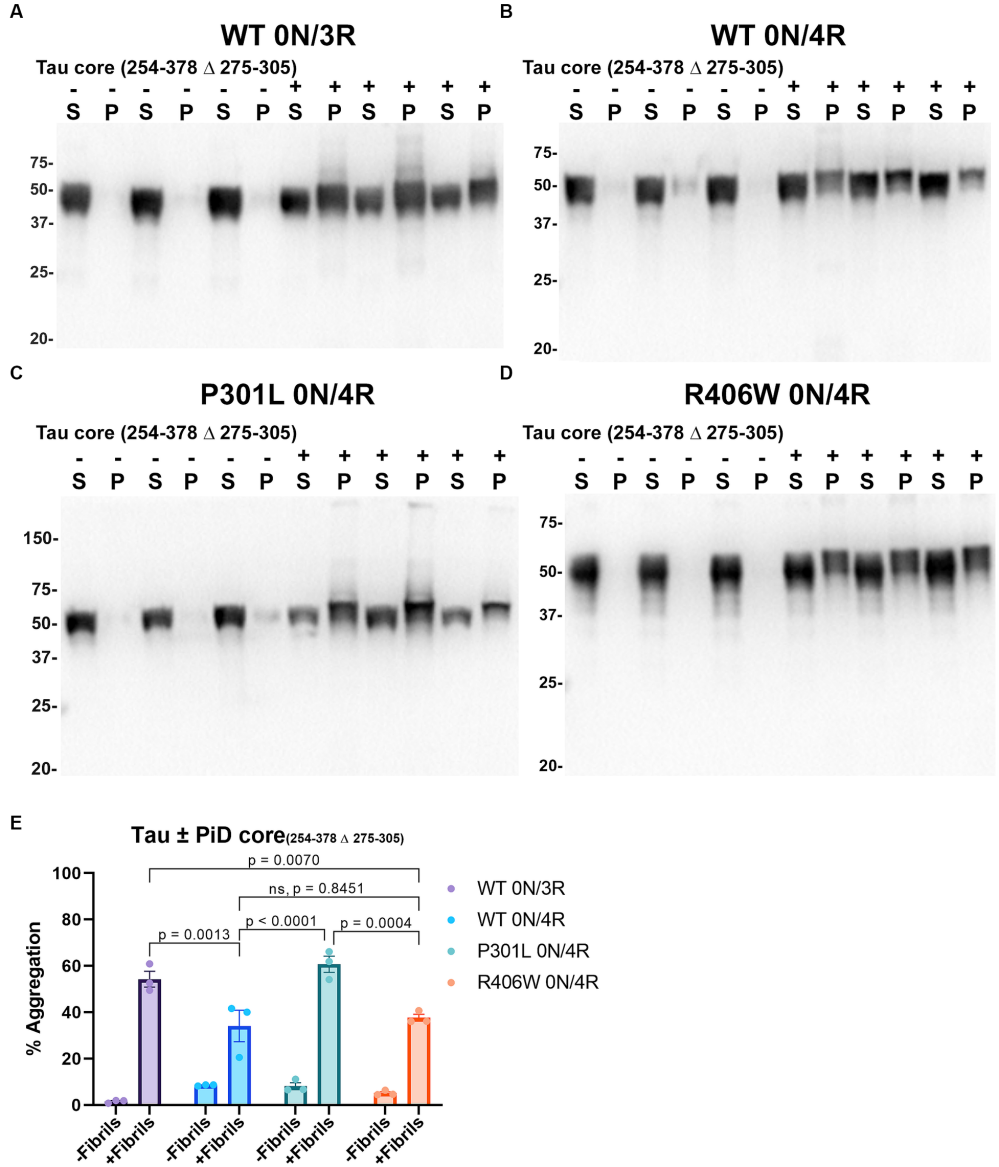
Immunoblot analyses of WT and Tau mutants seeded with the 254–378 Δ 275–305 PiD tau core fibrils in HEK293T cells. Western blots of lysate from HEK293T cells transfected to express **(A)** 0N/3R, **(B)** 0N/4R, **(C)** P301L 0N/4R, or **(D)** R406W 0N/4R human tau. Cells were untreated (−) or treated (+) with fibrils assembled from the tau fragments corresponding to amino acids 254–378 Δ 275–305 of full length 2 N/4R tau. Blots were probed with rabbit polyclonal 3026. ‘S’ indicates Triton soluble fractions and ‘P’ indicates Triton insoluble pellet fractions as indicated above each lane. The apparent mobilities of molecular weight markers are indicated on the left. **(E)** Percent aggregation was calculated using the formula [pellet/(pellet + supernatant)] × 100. *N* = 3 for each condition. Data are shown as the mean ± SEM. Two-way ANOVA was performed with correction for multiple comparisons using Tukey test. Statistically significant *p* values (*p* < 0.05) are indicated. ns indicates *p* value was not statistically significant.

Compared to PHFs and SFs observed in the brains of individuals with AD, the core element of filaments in the brains of individuals with PiD are comprised of residues K254-F378 of 3R tau isoforms which lack the 2nd repeat (R2; 275–305; numbering according to 2N/4R tau) and both narrow and wide filaments are observed by cryo-EM (Falcon et al., 2018a). In our assay, PiD tau core seeds were able to induce the aggregation of WT 0N/3R tau and WT, P301L and R406W 0N/4R tau, and was greater for WT 0N/3R tau and P301L 0N/4R tau compared to WT 0N/4R (Figure 6). The induced aggregation for WT and R406W 0N/4R tau was similar (Figure 6). We also observed a slight reduced shift in mobility of insoluble tau on SDS-PAGE, that can be associated with post-translational modifications such as phosphorylation (Lindwall and Cole, 1984). We examined this finding further by probing blots with phosphorylation specific antibodies 7F2 (pThr205) and PHF22 (pSer396/pSer404). Although Triton soluble tau is phosphorylated at these sites when cells are not treated with seeds, immunopositive bands around 50-55kD were detected in the Triton insoluble pellet for WT 0N/3R, WT, P301L and R406W 0N/4R tau when seeded with the PiD tau core with both 7F2 and PHF22 antibodies (Supplementary Figure S3). Seeds assembled from other tau cores such as tau core 306–380 and CBD tau core 274–380 were also more competent at inducing the aggregation of WT 0N/3R tau compared to WT 0N/4R (Supplementary Figures S4, S5). Although tau core 304–378 aggregates had detectable amyloid formation by K114 and ThT (Figure 2), they were not efficient at inducing the aggregation of WT 0N/3R or WT 0N/4R tau (Supplementary Figure S6). It should also be noted that WT 0N/3R, WT 0N/4R, P301L 0N/4R and R406W 0N/4R tau showed similar expression levels when expressed in HEK293T cells (Supplementary Figure S7).

### Seeds comprised of tau cores induce aggregation of tau with phosphorylation mimetics located in the proline rich or C terminus region of tau

**Figure 7:**
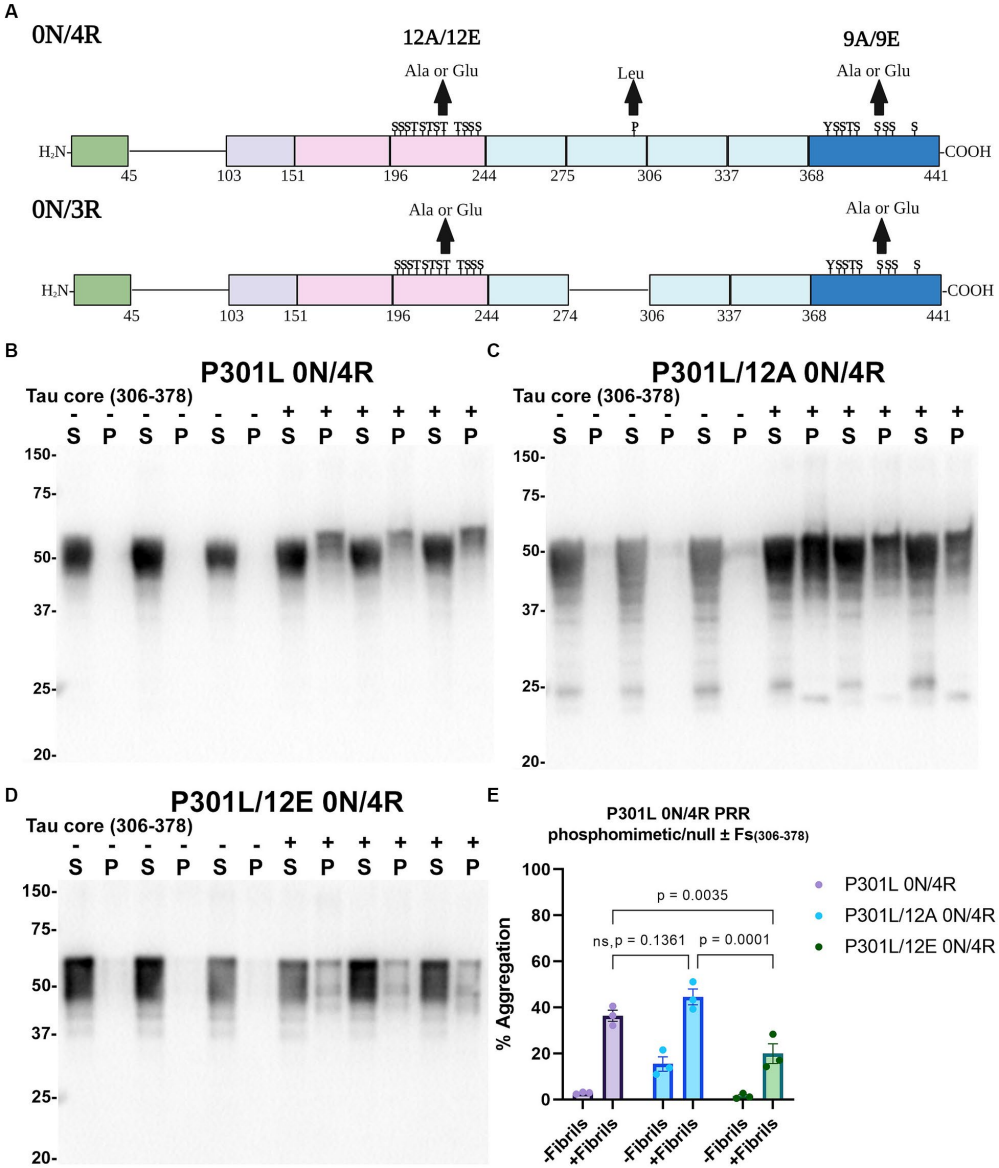
Immunoblot analyses of PRR phospho-mimetics and phospho-null in P301L 0N/4R tau seeded with AD tau core 306–378 fibrils in HEK293T cells. **(A)** Numbering is according to the longest tau isoform 2N/4R. Phospho-mimetic or phospho-null constructs were made by mutating threonine T, serine S, and tyrosine Y residues to glutamate E or alanine A, respectively. Created with BioRender.com. Western blots of lysate from HEK293T cells transfected to express **(B)** P301L 0N/4R, **(C)** P301L/12A 0N/4R, or **(D)** P301L/12E 0N/4R human tau. Cells were untreated (−) or treated (+) with fibrils assembled from the tau fragments corresponding to amino acids 306–378 of full length 2 N/4R tau. Blots were probed with rabbit polyclonal 3026. ‘Fs’ indicates fibrils. ‘S’ indicates Triton soluble fractions and ‘P’ indicates Triton insoluble pellet fractions as indicated above each. The apparent mobilities of molecular weight markers are indicated on the left. **(E)** Percent aggregation was calculated using the formula [pellet/(pellet + supernatant)] × 100. *N* = 3 for each condition. Data are shown as the mean ± SEM. Two-way ANOVA was performed with correction for multiple comparisons using Tukey test. Statistically significant *p* values (*p* < 0.05) are indicated. ns indicates *p* value was not statistically significant.

**Figure 8:**
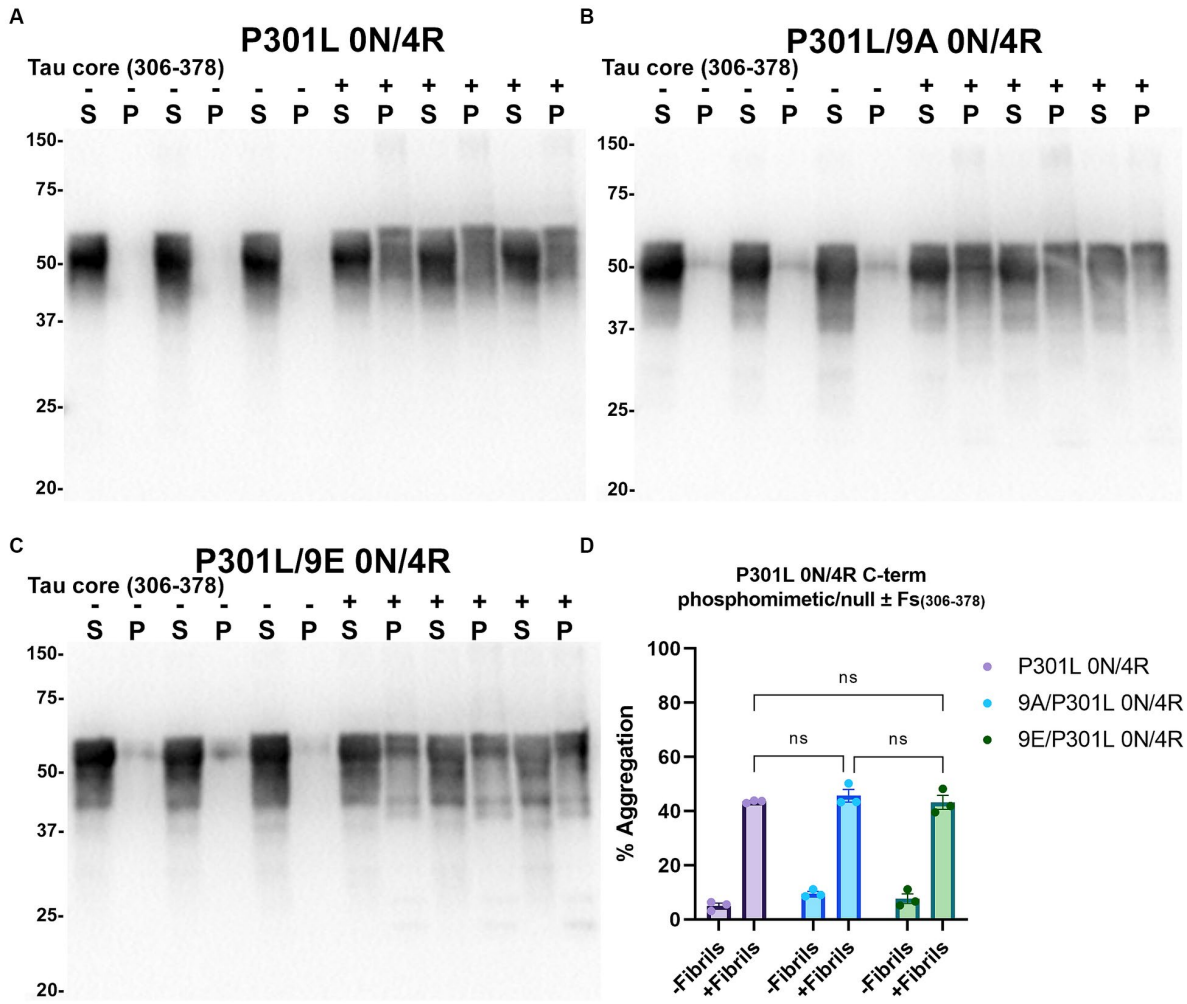
Immunoblot analyses of C-terminus phospho-mimetics and phospho-null in P301L 0N/4R tau treated with AD tau core 306–378 fibrils in HEK293T cells. Western blots of lysate from HEK293T cells transfected to express **(A)** P301L 0N/4R, **(B)** P301L/9A 0N/4R, or **(C)** P301L/9E 0N/4R human tau. Cells were untreated (−) or treated (+) with fibrils assembled from the tau fragments corresponding to amino acids 306–378 of full length 2N/4R tau. Blots were probed with rabbit polyclonal 3026. ‘Fs’ indicates fibrils. ‘S’ indicates Triton soluble fractions and ‘P’ indicates Triton insoluble pellet fractions as indicated above each. The apparent mobilities of molecular weight markers are indicated on the left. **(D)** Percent aggregation was calculated using the formula [pellet/(pellet + supernatant)] × 100. *N* = 3 for each condition. Data are shown as the mean ± SEM. Two-way ANOVA was performed with correction for multiple comparisons using Tukey test. ns indicates *p* value was not statistically significant.

**Figure 9:**
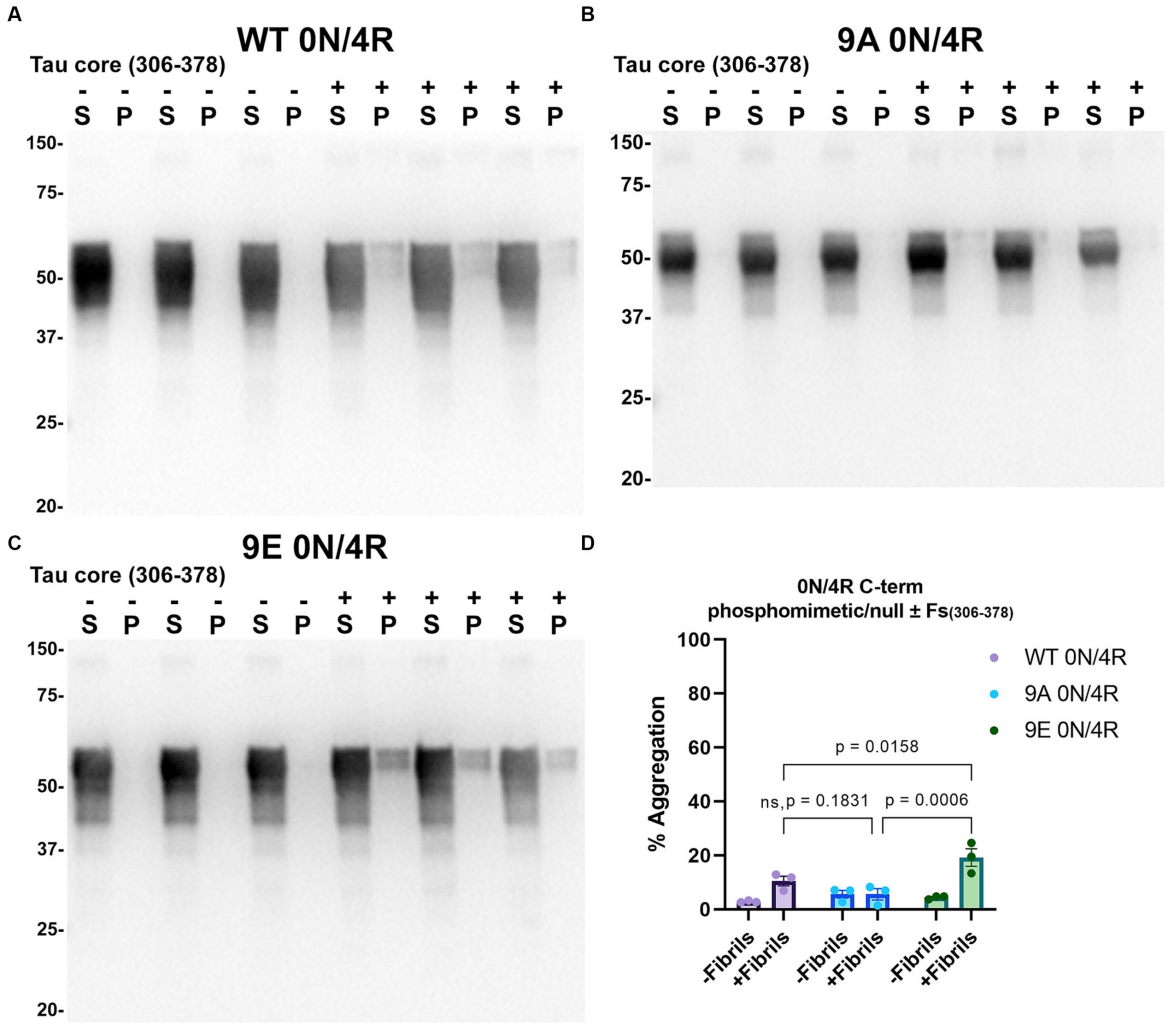
Immunoblot analyses of C-terminus phospho-mimetics and phospho-null in WT 0N/4R treated with AD tau core 306–378 fibrils. Western blots of lysate from HEK293T cells transfected to express **(A)** WT 0N/4R, **(B)** 9A 0N/4R or **(C)** 9E 0N/4R human tau. Cells were untreated (−) or treated (+) with fibrils assembled from the tau fragments corresponding to amino acids 306–378 of full length 2N/4R tau. Blots were probed with rabbit polyclonal 3026. ‘Fs’ indicates fibrils. ‘S’ indicates Triton soluble fractions and ‘P’ indicates Triton insoluble pellet fractions as indicated above each lane. The apparent mobilities of molecular weight markers are indicated on the left. **(D)** Percent aggregation was calculated using the formula [pellet/(pellet + supernatant)] × 100. *N* = 3 for each condition. Data are shown as the mean ± SEM. Two-way ANOVA was performed with correction for multiple comparisons using Tukey test. Statistically significant *p* values (*p* < 0.05) are indicated. ns indicates *p* value was not statistically significant.

**Figure 10:**
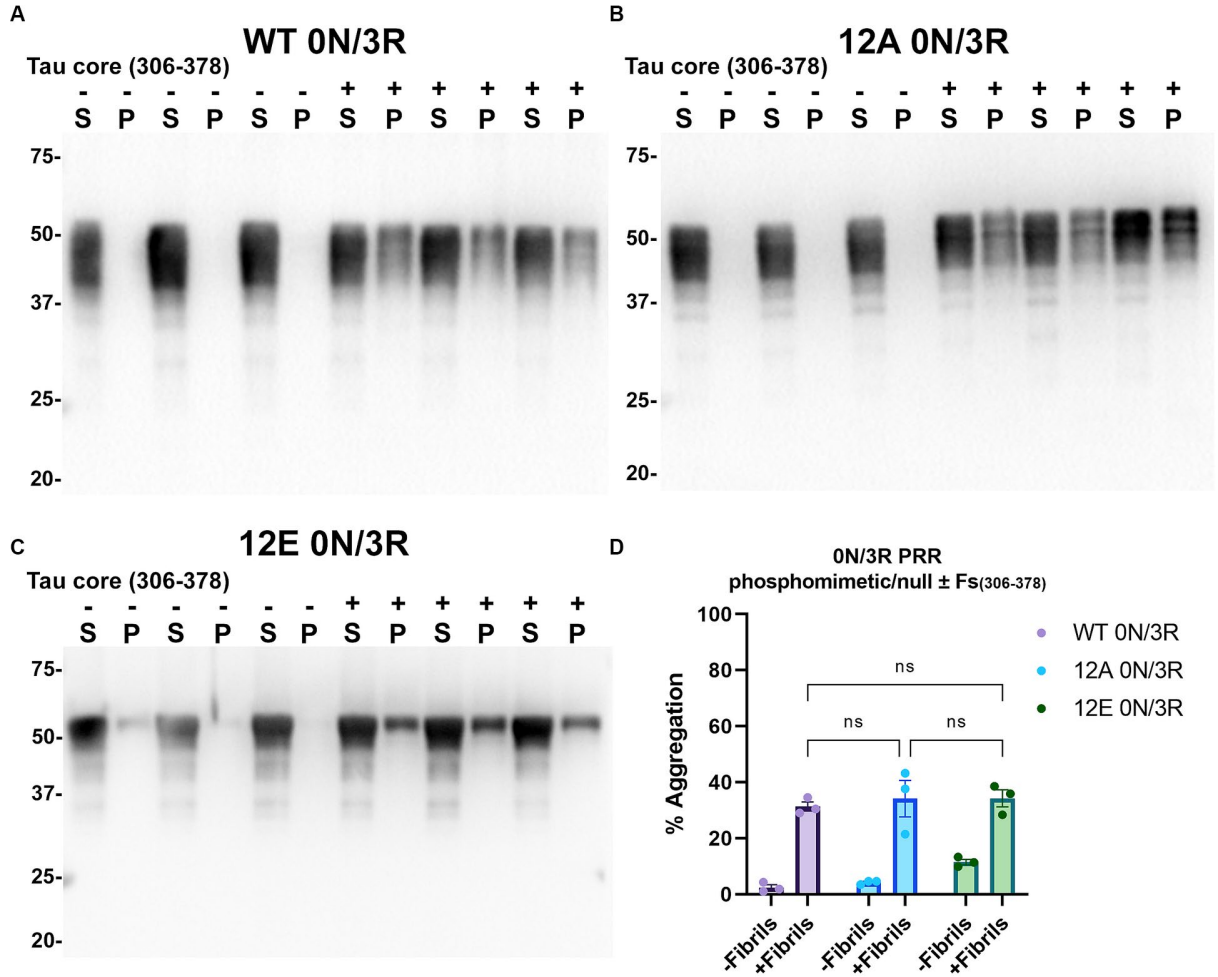
Immunoblot analyses of PRR phospho-mimetics and phospho-null in WT 0N/3R tau seeded with AD tau core 306–378 fibrils in HEK293T cells. Western blots of lysate from HEK293T cells transfected to express **(A)** WT 0N/3R, **(B)** 12A 0N/3R or **(C)** 12E 0N/3R human tau. Cells were untreated (−) or treated (+) with fibrils assembled from the tau fragments corresponding to amino acids 306–378 of full length 2N/4R tau. Blots were probed with rabbit polyclonal 3026. ‘Fs’ indicates fibrils. ‘S’ indicates Triton soluble fractions and ‘P’ indicates Triton insoluble pellet fractions as indicated above each lane. The apparent mobilities of molecular weight markers are indicated on the left. **(D)** Percent aggregation was calculated using the formula [pellet/(pellet + supernatant)] × 100. *N* = 3 for each condition. Data are shown as the mean ± SEM. Two-way ANOVA was performed with correction for multiple comparisons using Tukey test. ns indicates *p* value was not statistically significant.

**Figure 11:**
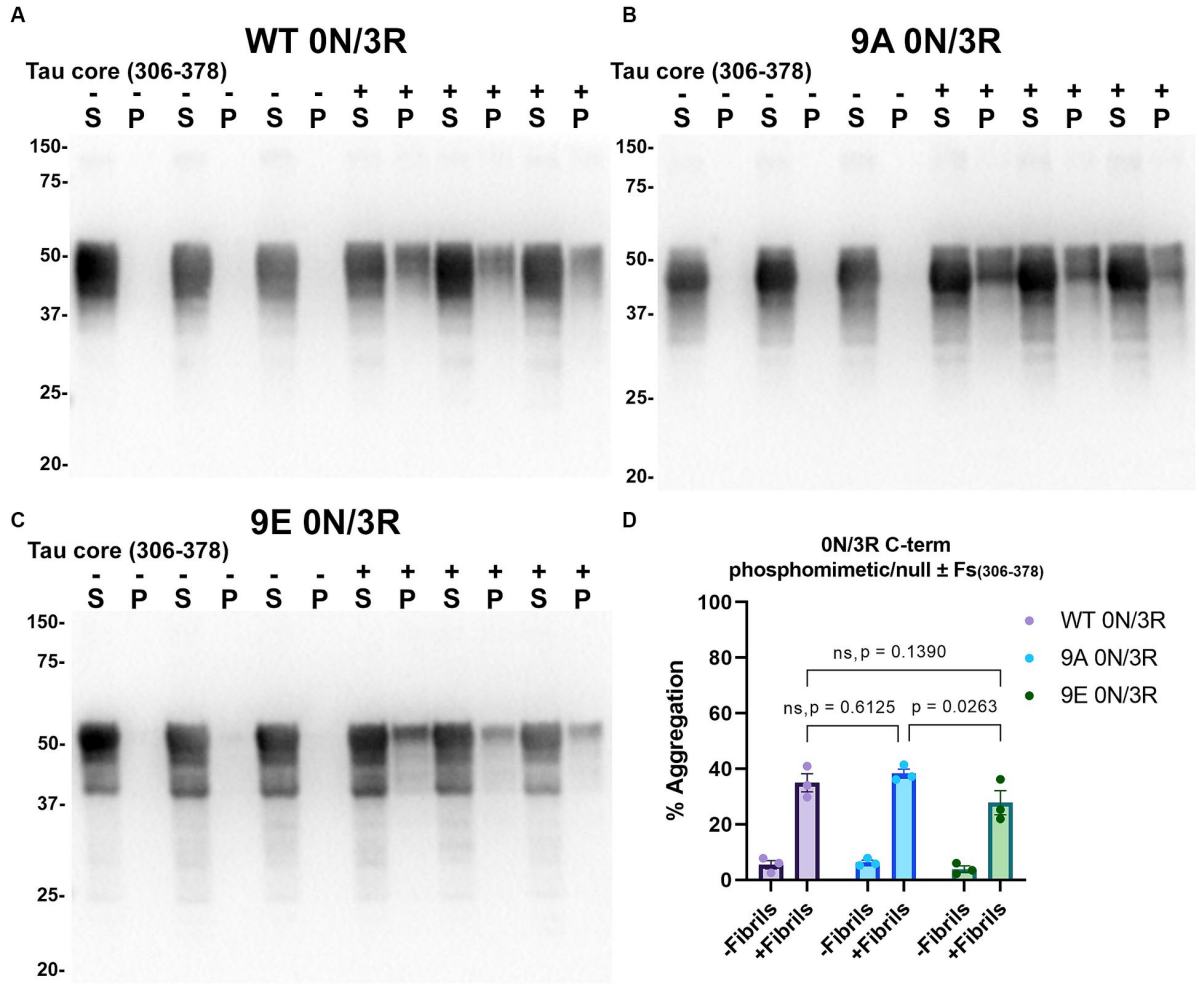
Immunoblot analyses of C-terminus phospho-mimetics and phospho-null in WT 0N/3R tau seeded with AD tau core 306–378 fibrils in HEK293T cells. Western blots of lysate from HEK293T cells transfected to express **(A)** WT 0N/3R, **(B)** 9A 0N/3R, or **(C)** 9E 0N/3R human tau. Cells were untreated (−) or treated (+) with fibrils assembled from the tau fragments corresponding to amino acids 306–378 of full length 2N/4R tau. Blots were probed with rabbit polyclonal 3026. ‘Fs’ indicates fibrils. ‘S’ indicates Triton soluble fractions and ‘P’ indicates Triton insoluble pellet fractions as indicated above each lane. The apparent mobilities of molecular weight markers are indicated on the left. **(D)** Percent aggregation was calculated using the formula [pellet/(pellet + supernatant)] × 100. *N* = 3 for each condition. Data are shown as the mean ± SEM. Two-way ANOVA was performed with correction for multiple comparisons using Tukey test **(D)**. Statistically significant *p* values (*p* < 0.05) are indicated. ns indicates *p* value was not statistically significant.

Tau (2N/4R) has 85 serine/threonine/tyrosine residues which are potential phosphorylation sites. These sites have been investigated *in vitro* and *in vivo* using phospho-mimetic and phospho-null mutant tau proteins to assess their propensity for seeding and aggregate formation (Waxman and Giasson, 2011; Gilley et al., 2016; Xia et al., 2020). Previous studies have identified sites in tau proteins that are phosphorylated in sarkosyl insoluble material under disease conditions such as AD (Wesseling et al., 2020) and many of these sites are located in the proline rich region (PRR) and the C terminus of tau (Figure 1). Phospho-mimetic and phospho-null constructs were generated by mutating Ser/Thr/Tyr to Glu (E) or Ala (A), respectively, to study these phosphorylation sites in the presence of AD tau core 306–378 at 12 sites in the PRR (designated 12E and 12A) and 9 sites in the C terminus (designated 9E and 9A) (Figure 7A). Based on the results above, we introduced these mutations in constructs expressing WT 0N/3R and P301L 0N/4R tau, which are both prone to seeding with tau core seeds, as well as WT 0N/4R tau which is more refractory to seeding. WT 0N/3R, WT 0N/4R and P301L 0N/4R tau do not tend to self-aggregate in the absence of exogenous fibrils in our cell-based assay and served as controls. In the absence of AD tau core seeds, phospho-mimetic and phospho-null tau displayed little tendency for self-aggregation (Figures 7–11), although some self-aggregation of 0N/4R P301L 12A was noted (P301L vs. P301L/12A, *p* = 0.02 and P301L/12A vs. P301L/12E, *p* = 0.0108) (Figure 7). P301L 0N/4R tau and P301L/12A 0N/4R tau seeded similarly when cells were treated with AD tau core 306–378 polymers (Figure 7). P301L/12E 0N/4R tau was also induced to aggregate but compared to P301L/12A 0N/4R tau and P301L 0N/4R tau there was less Triton insoluble material when treated with the AD tau core 306–378 seeds (Figure 7). P301L 0N/4R tau with phospho-mimetic or phospho-null sites in the C terminus (9A and 9E) also aggregated in the presence of AD tau core 306–378 seeds, but the findings were not significantly different from P301L 0N/4R tau (Figure 8). To further explore the seeding propensity of tau phospho-mutants in the context of WT tau, cells expressing WT 0N/4R, 9A 0N/4R, and 9E 0N/4R tau were treated with AD tau core 306–378 seeds. While WT 0N/4R and 9A 0N/4R displayed little aggregation, a significant increase in aggregation of 9E 0N/4R tau was observed (Figure 9). Similar studies with cells expressing WT 0N/3R, 12A 0N/3R, and 12E 0N/3R tau did not reveal any significant difference between WT and phosphorylation sites mutant proteins (Figure 10). Treatment of cells expressing 9A 0N/3R or 9E 0N/3R tau with AD tau core 306–378 seeds also induced tau aggregation, and there was more Triton insoluble tau formed with 9A 0N/3R tau compared to 9E 0N/3R tau, but neither were significantly different from WT 0N/3R tau (Figure 11).

## Discussion

Tau proteins abnormally accumulate into filamentous structures in neurons and glial cells in diseases known as tauopathies (Buée and Delacourte, 1999; Buée et al., 2000). There are over 20 tauopathies that have been characterized including AD, CBD, PiD, and frontotemporal dementia with parkinsonism-17 (FTDP-17) (Lee et al., 2001). Recent cryo-EM evidence has shown that the tau filaments that burden the CNS cell types are structurally distinct in their folding patterns and amino acid composition of their ordered cores (Scheres et al., 2020; Kovacs et al., 2022; Pinzi et al., 2023), which may suggest a potential explanation for the clinical and neuropathological heterogeneity observed between these diseases.

Since filaments from a patient with AD were first visualized by EM in 1963 (Kidd, 1963), they have been further described by their insolubility, abundant post-translational modifications, resistivity to protease/denaturants, and morphology, termed PHF and SF in AD, which are the units of the pathological NFTs, NTs, and DNs (Wischik et al., 1985; Crowther et al., 1989; Greenberg and Davies, 1990; Crowther, 1991). Moreover, the PHF core originated from the partial digestion of PHFs with pronase, resulting in a fragment within the MTBD of tau that remains structurally intact compared to the digested fuzzy coat comprising the N and C terminus (Wischik et al., 1988a; Crowther et al., 1989; Novak et al., 1993; von Bergen et al., 2006). A more complete description of these filaments has been elucidated by cryo-EM in which investigators have resolved the high-resolution atomic structures of the ordered cores of several tauopathies, including but not limited to, sporadic and inherited AD, CBD, PiD, progressive supranuclear palsy, and chronic traumatic encephalopathy (Fitzpatrick et al., 2017; Falcon et al., 2018a,b, 2019; Zhang et al., 2020; Goedert, 2021; Shi et al., 2021). More recently, it was shown that the AD, PiD, and CBD tau cores can fibrillize without commonly used cofactors such as heparin (Carlomagno et al., 2021).

While tau accumulation is a hallmark of these diseases, the mechanisms through which this occurs are still not completely established. Tau-related neurodegeneration has been hypothesized to arise through a prion-like mechanism in which a tau “seed,” through conformational templating, induces a conformation change in naïve tau which spreads in the brain through anatomically connected regions (Guo and Lee, 2011; Ayers et al., 2018). This seeding mechanism has been studied using truncated tau proteins composed of the R1–R4 (K18) and R1–R4 Δ R2 (275–305) (K19) (Guo and Lee, 2011; Strang et al., 2018; Xia et al., 2019, 2021) as well as full-length tau proteins (Guo and Lee, 2011). While tau proteins were first induced to polymerize *in vitro* with chemical polymers, filaments formed under these conditions have been shown to differ in their folding patterns compared to those from patient brains (Zhang et al., 2019). Seeding activity of tau has also been investigated *in vivo* using patient and mouse derived brain lysate (Clavaguera et al., 2009, 2013; He et al., 2020). While patient samples can perhaps more accurately replicate the plethora of tau conformers observed *in vivo*, the scarcity of human brain samples, interpatient variability, and other contaminating amyloidogenic proteins present significant challenges to the field. Although tau fibrils made from recombinant protein lack post-translational modifications such as phosphorylation, they offer a reliable and reproducible method for performing seeding experiments.

The tau core compositions resolved by cryo-EM from patients with tauopathies, while resulting in unique folds, minimally share the 3rd and 4th repeat of the MTBD. The MTBD has been extensively investigated not only for its physiological functions, but also for its ability to polymerize. Certain motifs in the MTBD, namely PHF6 (306VQIVYK311) and PHF6* (275VQIINK280), have been investigated in the context of tau aggregation kinetics (von Bergen et al., 2000; Goux et al., 2004; Li and Lee, 2006). Subsequently, tau cores used in this study and others that have been confirmed by cryo-EM, contain the PHF6 motif.

A current bottleneck of studying this mechanism is the ability to efficiently seed WT tau, while familial FTDP-17 mutations such as P301L or P301S are more prone to seeded aggregation and have been much more extensively studied (Strang et al., 2018). This is crucial as most tauopathies are sporadic and not downstream of missense mutations in the microtubule associated protein tau (MAPT) locus. Previous studies have shown modest accumulation of insoluble tau when attempting to seed full length WT tau isoforms using tau fibrils made from K18 (244–372) and K19 (244–372 Δ 275–305) (Strang et al., 2018; Xia et al., 2021), which do not exist physiologically, or full-length tau which resists fibrillization due to its high solubility and intrinsically disordered nature. Additionally, these fibrils are typically induced to aggregate with cofactors such as RNA, fatty acids, or the most widely used, heparin (Goedert et al., 1996; Kampers et al., 1996; Wilson and Binder, 1997). The exact mechanism for how heparin facilitates tau fibrillization is not known, but it likely influences conformation by affecting the overall charge of tau allowing for more accessible stacking of tau molecules. Additionally, investigations of the mechanism by which heparin affects tau fibril internalization have found that the presence of heparin is inhibitory in heparin sulfate proteoglycan receptor mediated uptake (Holmes et al., 2013), which presents as a potential confound in seeding experiments when using fibrils made in the presence of heparin.

Our study sought to characterize the fibrillization kinetics and seeding activity of the AD, PiD, and CBD tau cores in a cell culture-based aggregation assay, and to investigate the differences in the seeding propensity of 2 tau isoforms (0N/4R and 0N/3R) and FTDP-17 associated mutations (P301L and R406W). These two disease-variants share decreased affinity for MTs compared to WT (Xia et al., 2019), while the P301L mutation but not R406W is significantly more vulnerable to seeded aggregation (Guo and Lee, 2011; Strang et al., 2019a). Additionally, 3R tau isoforms also exhibit decreased affinity for MTs compared to 4R (Goedert and Jakes, 1990; Xia et al., 2021), lending credence to the hypothesis that MT dissociated tau is perhaps causal for aggregation.

Tauopathies are further defined by the isoform abundance in their inclusions; specifically, AD aggregates contain both 3R and 4R tau (Goedert et al., 1989a; Jakes et al., 1991), PiD is mostly 3R tau (De Silva et al., 2003), and tau inclusions in CBD are mostly 4R tau (Sergeant et al., 1999). We observed marked differences between aggregation propensity between 3R and 4R tau isoforms when seeding with AD, PiD, and CBD tau cores. Most of the AD tau cores were able to seed WT 0N/3R tau more than WT 0N/4R tau (Figures 4, 5). The PiD tau core was able to seed WT 0N/3R tau more than WT 0N/4R tau (Figure 6), which predominantly accumulates 3R positive tau aggregates (De Silva et al., 2003). Using cell-free aggregation assays, seeding barriers have been observed in the context of 3R and 4R tau isoforms using K19 and K18 (Dinkel et al., 2011; Kumar and Udgaonkar, 2018), in which 4R tau fibrils were less effective at seeding 3R tau monomer, which is at odds with our cell-seeding results using the CBD tau core (Supplementary Figure S5), despite pathological inclusions in CBD predominantly being 4R tau positive (Sergeant et al., 1999; Kouri et al., 2011). These studies also showed that 4R tau monomer is able to be seeded by 4R tau fibrils which was not robust in our cell aggregation assay. While this is surprising, additional post-translational modifications such as ubiquitination and acetylation have been investigated by cryo-EM and mass spectrometry and have been shown to occur on residues within the protofilament core (Arakhamia et al., 2020). These modifications likely influence fibril formation which perhaps cannot be replicated in an *in vitro* system. A limitation of our study is that relative expression levels of tau isoforms and mutants in the presence and absence of seeds were not compared, which might partially influence the seeding of tau proteins. Furthermore, we performed our seeding studies using a single amount of the polymerized tau core fragments. Additionally, while cryo-EM has shown that these protofilament cores from patients with AD, PiD, and CBD consist of the various amino acid sequences used in our study, we did not determine by cryo-EM if these tau cores fold similarly as those found in patient brains.

The mechanism(s) by which prion-like tau seeds become internalized by cells and lead to the intracellular aggregation of naïve tau is still not fully understood. Some hypotheses that have been investigated using cellular systems indicate that tau can be taken up by cells through endocytosis (Guo and Lee, 2011; Wu et al., 2013) or by exosomes (Wang et al., 2017). While endocytosis related pathways are a possible mechanism to explain the induction of cellular tau aggregation by exogenous tau core fibrils observed in our cell-based assay, it should be noted that the use of transfection reagents likely influences the ability for fibrils to enter the cell.

Under physiological conditions, tau facilitates the polymerization of MTs and regulates MT dynamics (Weingarten et al., 1975; Witman et al., 1976). Although tau proteins are highly soluble and intrinsically disordered with little secondary structure (Jeganathan et al., 2008b), they possibly adopt conformation(s) in solution such as a “paperclip conformation” (Jeganathan et al., 2006) or others that generate the MC1 and Alz-50 epitope, which were characterized using recombinant tau (Jicha et al., 1997). Although tau does not show tendency for aggregation, under conditions which have not been fully elucidated, tau can misfold, become hyperphosphorylated, and aggregate into heterogenous structures visualized histopathologically (Buée et al., 2000), the order of these events is still not completely understood. While tau accumulation is universally characteristic of these diseases, the morphology, isoform composition, and folding often allow us to differentiate between diseases. The PTM profile of tau aggregates from AD has been extensively investigated by immunohistochemistry using phosphorylation specific antibodies and mass spectrometry. Phosphorylation of tau protein is a PTM highly abundant during disease (Wesseling et al., 2020), and is also associated with stages of tangle formation histologically (Bancher et al., 1989; Xia et al., 2020; Moloney et al., 2022). It has been hypothesized that tau hyperphosphorylation mediates dissociation from MTs and promotes aggregation and filament formation. An alternative observation has been reported in that several studies have shown that certain phosphorylation sites, while showing a reduction in tau bound to MT or MT formation, are inhibitory toward aggregation (Schneider et al., 1999; Haj-Yahya et al., 2020). Indeed, tau cores used in our study, K18, K19, or even full-length tau proteins, do not require phosphorylation, and is therefore not a prerequisite for fibril formation to occur, further complicating the relationship between phosphorylation and aggregate formation.

In our study, we used phospho-mimetic and phospho-null constructs to investigate this association in both the PRR and the C-terminus region of tau. Likewise, previous studies have used phospho-mimetic and null mutations to study AD-like changes such as filament formation, insolubility, and effects on MT dynamics (Eidenmuller et al., 2000, 2001; Jeganathan et al., 2008a; Waxman and Giasson, 2011; Gilley et al., 2016; Xia et al., 2020). The constructs that we used targeted a large series (12 in the PRR region and 9 in the C-terminal region) of tau phosphorylation sites that coincide with pathogenic tau aggregation in human disease (Wesseling et al., 2020). We hypothesized that constitutive phosphorylation using phospho-mimicking mutations might promote seeding induced aggregation. Our results suggest that mimicking phosphorylation in the PRR at these sites, in addition to the P301L mutation appear to be less permissive for aggregation in the presence of seeds and perhaps may be inhibitory (Figure 7). On the other hand, C-terminal phospho-mimetic mutations in the presence of P301L did not affect seeding-induced aggregation (Figure 8), suggesting the addition of the P301L mutation is perhaps less compatible with structural changes induced by phosphorylation sites in the PRR versus the C-terminus. Furthermore, the C-terminus phospho-mimetic sites in the context of WT 0N/4R tau with seeding exhibited modest increased aggregation (Figure 9). Interestingly phospho-mimetic and phospho-null alterations within the PRR of 0N/3R tau showed no difference in Triton insolubility patterns compared to WT 0N/3R tau (Figure 10) further suggesting that tau isoforms have different susceptibilities to seeding and responses to phosphorylation. Certain phospho-mimetic sites have been found to prevent aggregation (Strang et al., 2019b), while others enhance aggregation (Xia et al., 2020). The presence of phospho-mimetic clusters in the PRR has been shown to affect microtubule polymerization (Eidenmuller et al., 2001), while a single phospho-mimetic site in the PRR has been shown to have enhanced binding of MTs (Xia et al., 2020). The effects of phosphorylation are not straightforward and some sites have been shown to affect tau folding (Jeganathan et al., 2008a) and MT dynamics (Kiris et al., 2011) quite differently compared to when they are paired with distal sites. These findings suggest that certain tau PTMs likely exhibit heterogenous contributions to aggregate formation, which are also highly affected by tau MTBD composition. While the use of phospho-mimetics allows us to study the effects of sustained phosphorylation, it should be noted that while the substitution of a Ser/Thr/Tyr with a glutamate mimics a phosphorylated residue due to the presence of a negative charged side chain at physiologic cellular conditions, it does not fully recapitulate the 2 negative charges and size of an authentic phosphate group. Overall, the impact of the phosphorylation mutants on tau seeded aggregation with the tau core polymers was in most conditions contrary to our expectation, supporting the notion that these phosphorylation events in human disease might occur after aggregation is initiated and therefore have limited impact on prion-like propagation. However, as tau phosphorylation can reduce tau-MT binding and assembly (Lindwall and Cole, 1984; Alonso et al., 1994), it is likely that it can regulate the amount of free tau that is available to be seeded. This hypothesis will need to be further explored in additional studies investigating tau-MT binding under seeded conditions.

Our study highlights the importance of initiator seed composition and the role of specific tau core fibrils in revealing isoform and mutant-specific changes in seeding propensity. Isoform or mutation-specific seeding effects were observed with AD, PiD, and CBD tau cores. Collectively, our studies indicate that WT 0N/4R tau was less susceptible to seeding than WT 0N/3R tau and P301L tau, but the underlying mechanism will need to be further investigated. While 6 tau isoforms are present in the adult human brain, it remains unknown what the individual contribution of, and interactions between, each isoform are in the context of seeding. Other PTM related influences on fibril formation and aggregation propensity will also have to be investigated to further clarify the role of phosphorylation and aggregate formation. Lastly, these structurally-ordered tau cores found in the brains of individuals with distinct tauopathies will offer a more physiologically relevant alternative in seeding studies, and can perhaps be used to distinguish tauopathies.

## Data availability statement

The raw data supporting the conclusions of this article will be made available by the authors, without undue reservation.

## Author contributions

GP: Formal analysis, Investigation, Writing – original draft, Writing – review & editing. BB: Investigation, Writing – review & editing. AR-D: Investigation, Writing – review & editing. ML: Investigation, Writing – review & editing, Funding acquisition. BG: Funding acquisition, Investigation, Writing – review & editing, Conceptualization, Formal analysis, Supervision, Writing – original draft.

## Glossary

3R: 3-repeat
4R: 4-repeat
AD: Alzheimer’s disease
PiD: Pick’s disease
CBD: Corticobasal degeneration
CNS: central nervous system
DNs: dystrophic neurites
EM: electron microscopy
FBS: fetal bovine serum
FTDP-17: frontotemporal dementia with parkinsonism-17
MT: microtubule
MTBD: microtubule binding domain
NFTs: neurofibrillary tangles
NTs: neuropil threads
PHFs: paired helical filaments
PRR: proline rich region
SFs: straight filaments
TBS: Tris-buffered saline
ThT: Thioflavin-T
