# Supplementary material for: Polymerization of recombinant tau core fragments in vitro and seeding studies in cultured cells
Source: Front Neurosci. 2023 Dec 15;17:1268360. doi: 10.3389/fnins.2023.1268360 (PMC10757379; doi:10.3389/fnins.2023.1268360)
Supplement: Supplementary file 1 [file Data_Sheet_1.pdf]

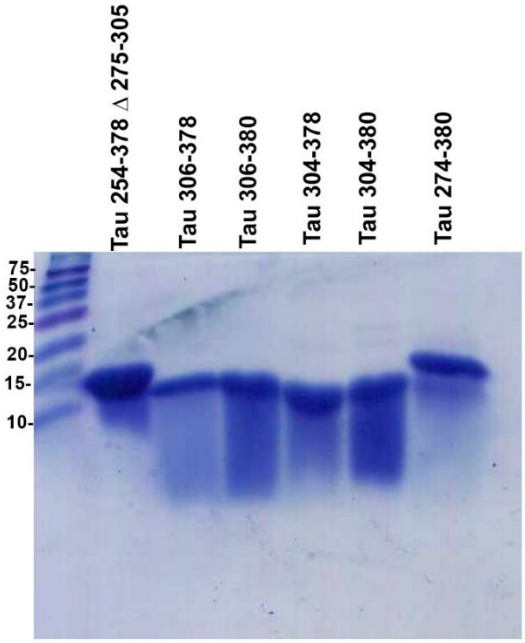

Supplemental Figure 1. Coomassie R-250 stained SDS polyacrylamide gel of purified recombinant human tau fragments. Purified tau fragments as labeled above each lane (5 μg) were resolved on a 15% polyacrylamide gel by SDS-PAGE and stained with Coomassie R-250 followed by de-staining. Labelled molecular mass marker is located on the left of the gel.

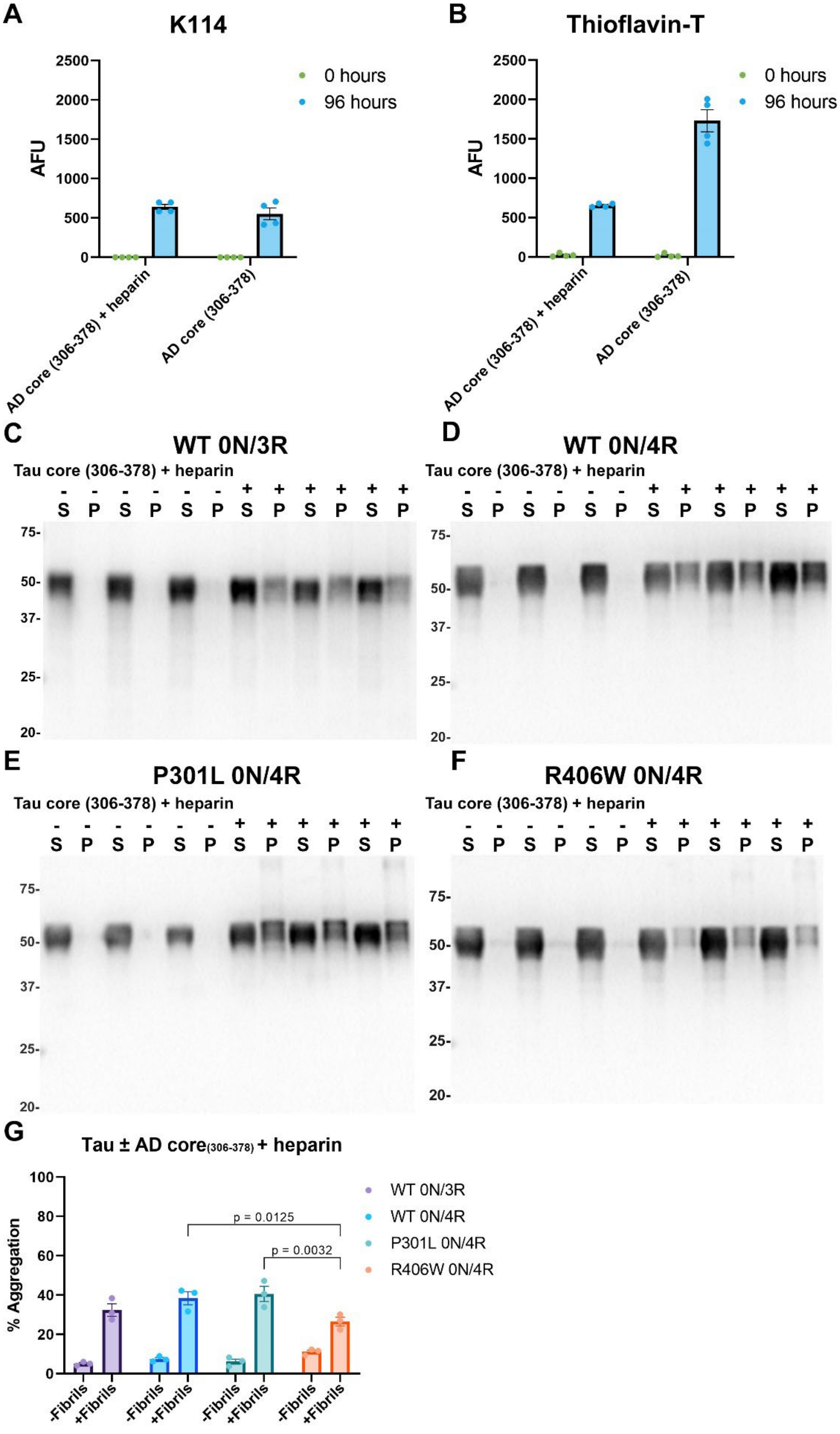
A
K114
AFU
2500
2000
1500
1000
500
0
0 hours
96 hours
AD core (306-378) + heparin
AD core (306-378)
B
Thioflavin-T
AFU
2500
2000
1500
1000
500
0
0 hours
96 hours
AD core (306-378) + heparin
AD core (306-378)
C
WT 0N/3R
Tau core (306-378) + heparin
- - - - - - + + + + + +
S P S P S P S P S P S P
75-
50-
37-
25-
20-
D
WT 0N/4R
Tau core (306-378) + heparin
- - - - - - + + + + + +
S P S P S P S P S P S P
75-
50-
37-
25-
20-
E
P301L 0N/4R
Tau core (306-378) + heparin
- - - - - - + + + + + +
S P S P S P S P S P S P
75-
50-
37-
25-
20-
F
R406W 0N/4R
Tau core (306-378) + heparin
- - - - - - + + + + + +
S P S P S P S P S P S P
75-
50-
37-
25-
20-
G
Tau ± AD core(306-378) + heparin
% Aggregation
100
80
60
40
20
0
p = 0.0125
p = 0.0032
WT 0N/3R
WT 0N/4R
P301L 0N/4R
R406W 0N/4R
-Fibrils
+Fibrils
-Fibrils
+Fibrils
-Fibrils
+Fibrils
-Fibrils
+Fibrils

Supplemental Figure 2. Immunoblot analyses comparing the seeding activity of heparin induced AD tau core 306-378 fibrils in HEK293T cells. **(A)** K114 and **(B)** ThT fluorometry were used to monitor amyloid formation. Measurements were taken at 0 and 96 hours for tau core 306-378 fibrils in the presence and absence of heparin. Data are shown as mean ± SEM. Western blots of lysate from HEK293T cells transfected to express **(C)** WT 0N/3R**,** **(D)** WT 0N/4R, **(E)** P301L 0N/4R, or **(F)** R406W 0N/4R human tau. Cells were untreated (-) or treated (+) with fibrils comprised of the tau fragment corresponding to amino acids 306-378, numbers according to full length 2N/4R tau, polymerized in the presence of heparin. Blots were probed with rabbit polyclonal 3026. **'**S' indicates Triton soluble fractions and 'P' indicates Triton insoluble pellet fractions as indicated above each lane. The apparent mobilities of molecular weight markers are indicated on the left. **(G)** Percent aggregation was calculated using the formula [pellet/(pellet + supernatant)] x 100. N=3 for each condition. Data are shown as the mean ± SEM. Two-way ANOVA was performed with correction for multiple comparisons using Tukey test **(G)**. Statistically significant p values ($p < 0.05$) are indicated.

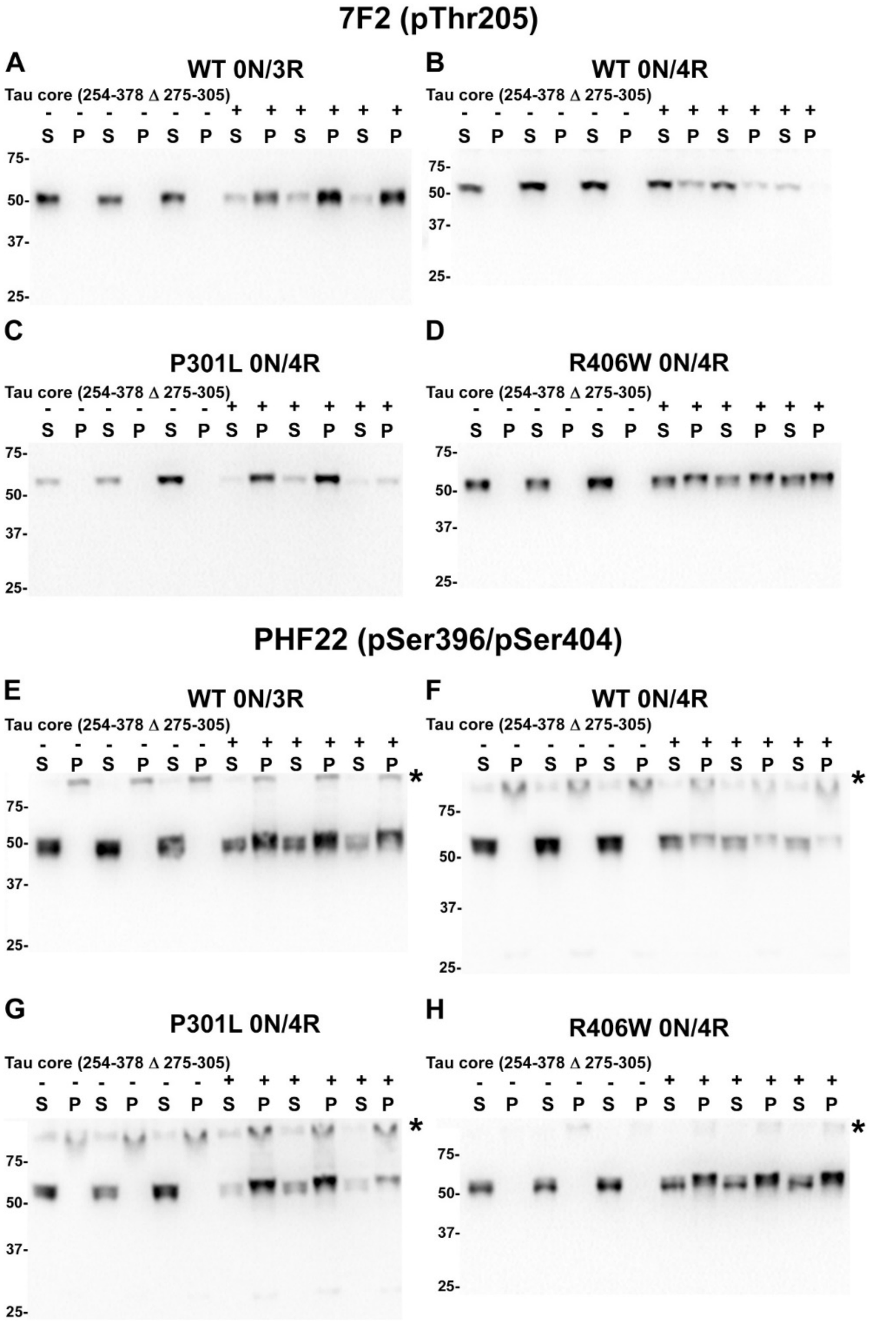

Supplemental Figure 3. Immunoblot analyses demonstrating phosphorylated tau in insoluble fraction induced when seeded with 254-378 Δ 275-305 PiD tau core fibrils. Western blots of lysate from HEK293T cells transfected to express **(A, E)** WT 0N/3R, **(B, F)** WT 0N/4R, **(C, G)** P301L 0N/4R, or **(D, H)** R406W 0N/4R human tau. Cells were untreated (-) or treated (+) with fibrils assembled from 254-378 Δ 275-305 tau fragment corresponding to numbering of full length 2N/4R tau. N=3 for each condition. 'S' indicates Triton soluble fractions and 'P' indicates Triton insoluble pellet fractions as indicated above each lane. The apparent mobilities of molecular weight markers are indicated on the left. Blots were probed with tau antibodies specific for phosphorylation at sites pT205 (7F2) and pS396/pS404 (PHF22). Asterisks indicate nonspecific band.

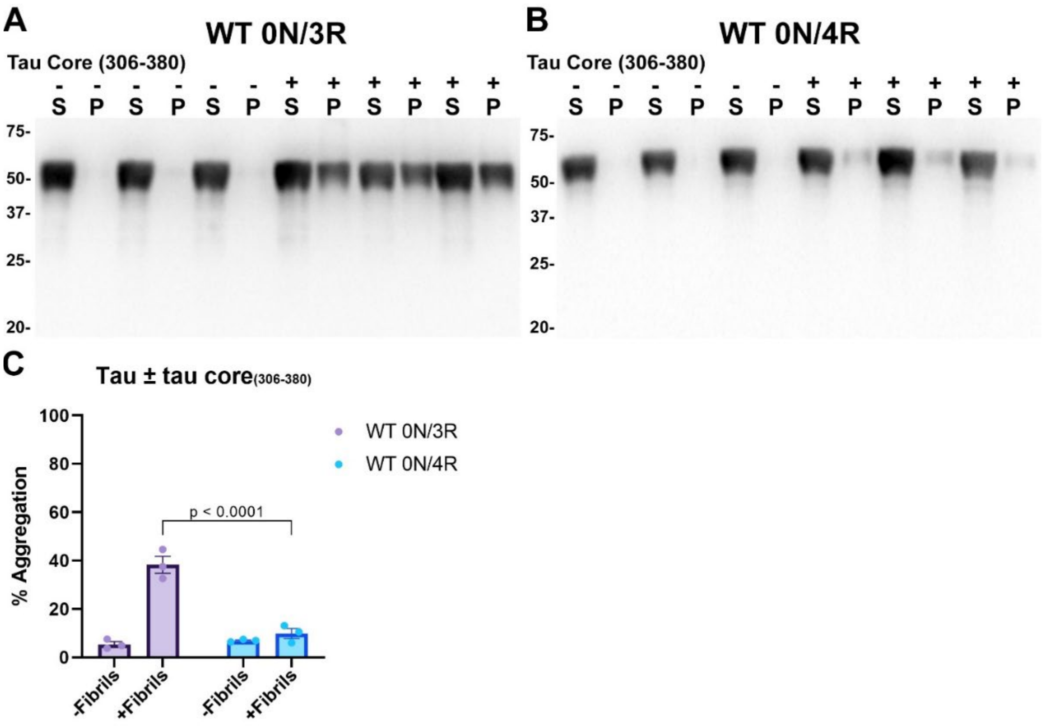

Supplemental Figure 4. Immunoblot analyses of WT Tau seeded with tau core 306-380 tau fibrils in HEK293T cells. Western blots of lysate from HEK293T cells transfected to express **(A)** WT 0N/3R or **(B)** WT 0N/4R human tau. Cells were untreated (-) or treated (+) with fibrils assembled from the tau fragments corresponding to amino acids 306-380 of full length 2N/4R tau**.** Blots were probed with rabbit polyclonal 3026. **'**S' indicates Triton soluble fractions and 'P' indicates Triton insoluble pellet fractions as indicated above each lane. The apparent mobilities of molecular weight markers are indicated on the left. (**C**) Percent aggregation was calculated using the formula [pellet/(pellet + supernatant)] x 100. N=3 for each condition. Data are shown as the mean ± SEM. Two-way ANOVA was performed with correction for multiple comparisons using Šidák test **(C)**. Statistically significant p value ($p < 0.05$) is indicated.

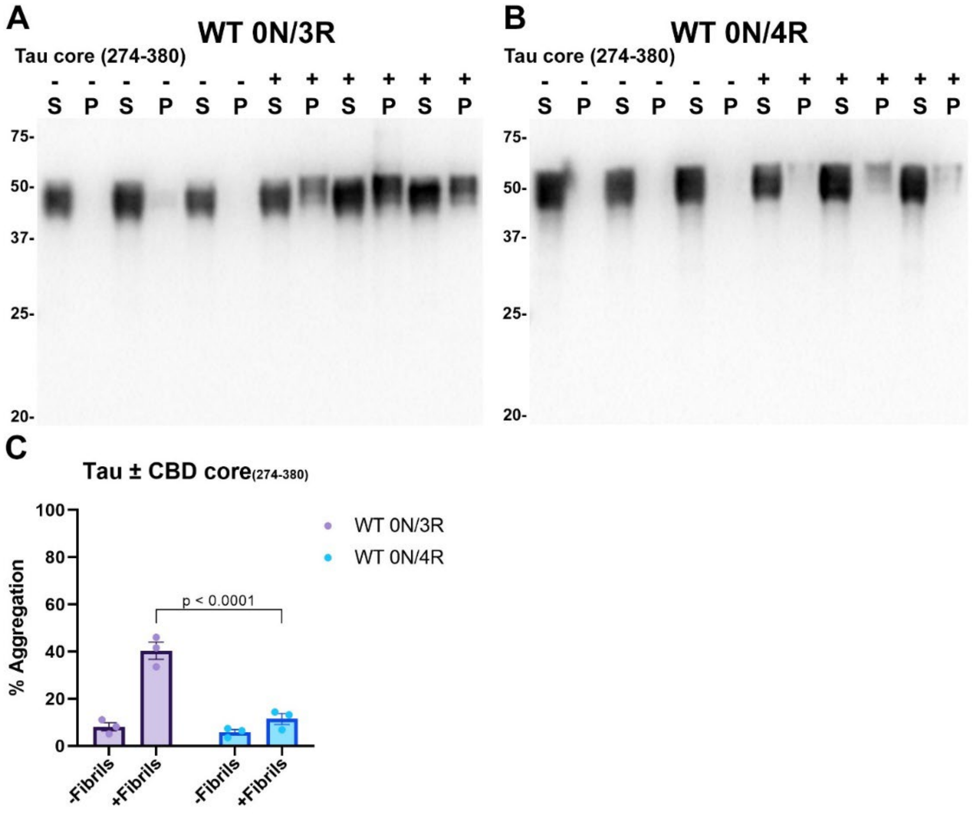

Supplemental Figure 5. Immunoblot analyses of WT Tau seeded with CBD tau core 274-380 fibrils in HEK293T cells. Western blots of lysate from HEK293T cells transfected to express **(A)** WT 0N/3R or **(B)** WT 0N/4R human tau. Cells were untreated (-) or treated (+) with fibrils assembled from the tau fragments corresponding to amino acids 274-380 of full length 2N/4R tau**.** Blots were probed with rabbit polyclonal 3026. **'**S' indicates Triton soluble fractions and 'P' indicates Triton insoluble pellet fractions as indicated above each lane. The apparent mobilities of molecular weight markers are indicated on the left. (**C**) Percent aggregation was calculated using the formula [pellet/(pellet + supernatant)] x 100. N=3 for each condition. Data are shown as the mean ± SEM. Two-way ANOVA was performed with correction for multiple comparisons using Šidák test. Statistically significant p value ($p < 0.05$) is indicated.

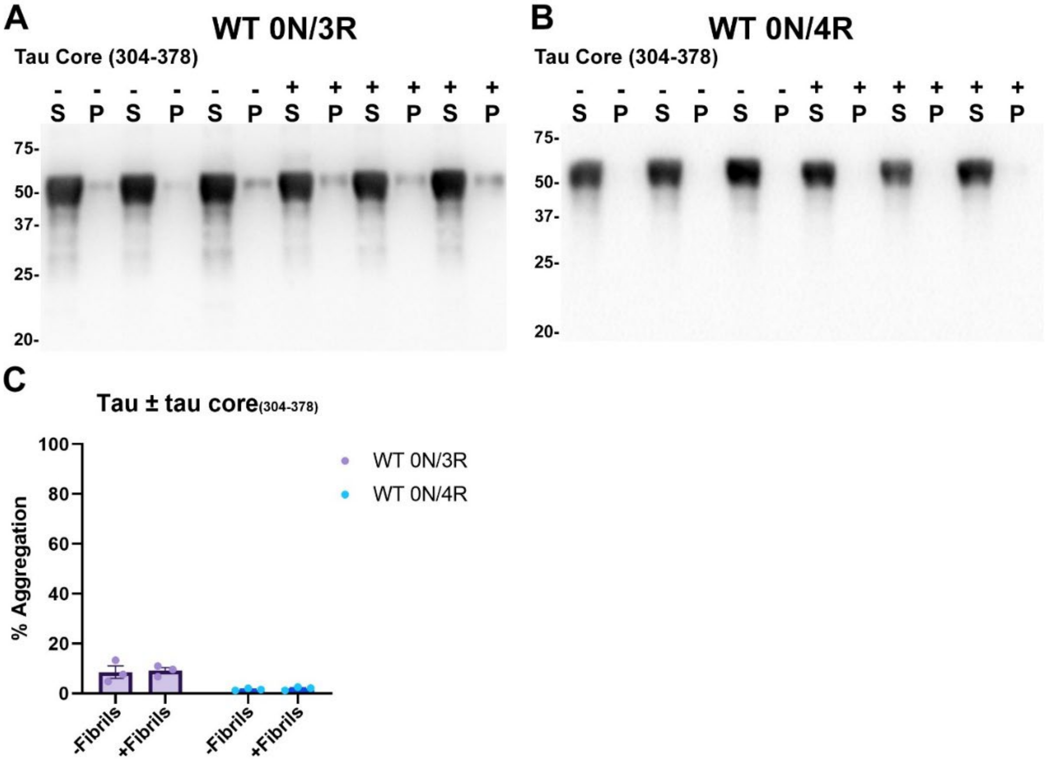

Supplemental Figure 6. Immunoblot analyses of WT Tau seeded with tau core 304-378 fibrils. Western blots of lysate from HEK293T cells transfected to express **(A)** WT 0N/3R or **(B)** WT 0N/4R human tau. Cells were untreated (-) or treated (+) with fibrils assembled from the tau fragments corresponding to amino acids 304-378 of full length 2N/4R tau**.** Blots were probed with rabbit polyclonal 3026. 'S' indicates Triton soluble fractions and 'P' indicates Triton insoluble pellet fractions as indicated above each lane. The apparent mobilities of molecular weight markers are indicated on the left. (**C**) Percent aggregation was calculated using the formula [pellet/(pellet + supernatant)] x 100. N=3 for each condition. Data are shown as the mean ± SEM.

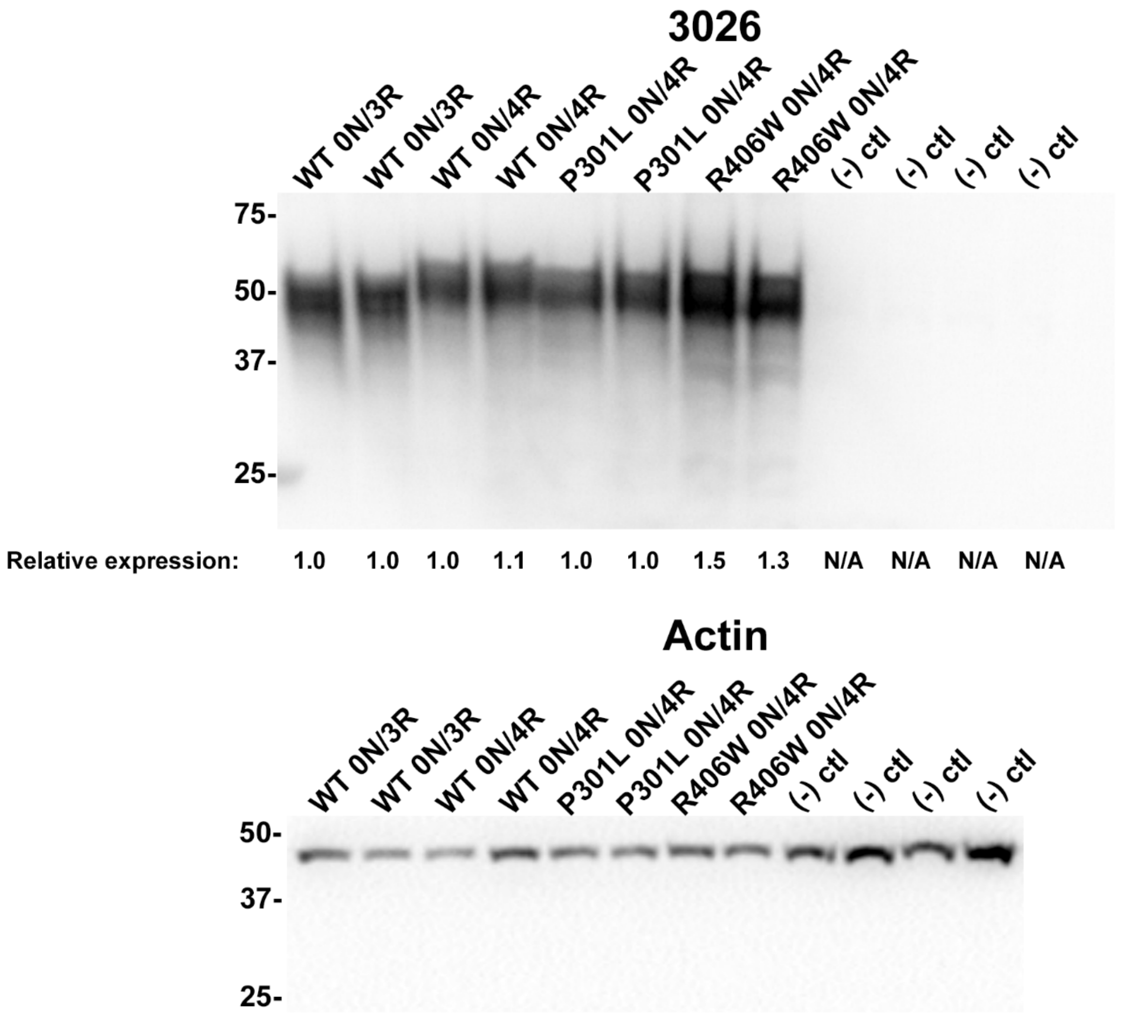

Supplemental Figure 7. Relative expression levels of WT Tau isoforms and mutants. HEK293T cells were transfected to express WT 0N/3R (N=2), WT 0N/4R (N=2), P301L 0N/4R (N=2) or R406W 0N/4R (N=2) human tau, or were untransfected as negative (-) controls (ctl)(N=4). Cell lysates were prepared by extracting in SDS sample buffer and analyzed by immunoblotting. Equal volumes were loaded and blots were probed with rabbit anti-tau antibody 3026 or an anti-actin antibody as a loading control. Quantification under the 3026 immunoblot was performed using ImageJ software and is expressed as relative signal compared to the first lane (WT 0N/3R).

Figure	Condition	Tau core	p value (+Fibrils vs -Fibrils)	Summary
4	WT 0N/3R	306-378	0.0009	***
4	WT 0N/4R	306-378	0.0193	*
4	P301L 0N/4R	306-378	0.0164	*
4	R406W 0N/4R	306-378	0.0003	***
5	WT 0N/3R	304-380	0.0199	*
5	WT 0N/4R	304-380	0.0197	*
5	P301L 0N/4R	304-380	0.0007	***
5	R406W 0N/4R	304-380	<0.0001	****
6	WT 0N/3R	254-378 Δ (275-305)	0.0001	***
6	WT 0N/4R	254-378 Δ (275-305)	0.0199	*
6	P301L 0N/4R	254-378 Δ (275-305)	0.0002	***
6	R406W 0N/4R	254-378 Δ (275-305)	<0.0001	****
7	P301L 0N/4R	306-378	0.0002	***
7	P301L/12A 0N/4R	306-378	0.0034	**
7	P301L/12E 0N/4R	306-378	0.0133	*
8	P301L 0N/4R	306-378	<0.0001	****
8	P301L/9A 0N/4R	306-378	0.0001	***
8	P301L/9E 0N/4R	306-378	0.0003	***
9	WT 0N/4R	306-378	0.0131	*
9	9A 0N/4R	306-378	0.9935	ns
9	9E 0N/4R	306-378	0.0109	*
10	WT 0N/3R	306-378	0.0001	***
10	12A 0N/3R	306-378	0.0102	*
10	12E 0N/3R	306-378	0.0021	**
11	WT 0N/3R	306-378	0.0012	**
11	9A 0N/3R	306-378	<0.0001	****
11	9E 0N/3R	306-378	0.0058	**
Fig.S2	WT 0N/3R	306-378 + Heparin	0.0011	**
Fig.S2	WT 0N/4R	306-378 + Heparin	0.0008	***
Fig.S2	P301L 0N/4R	306-378 + Heparin	0.001	**
Fig.S2	R406W 0N/4R	306-378 + Heparin	0.003	**
Fig. S4	WT 0N/3R	306-380	0.0009	***
Fig. S4	WT 0N/4R	306-380	0.2238	ns
Fig. S5	WT 0N/3R	274-380	0.0014	**
Fig. S5	WT 0N/4R	274-380	0.0937	ns

Supplemental Table 1. Statistical analyses of seed treated compared to unseeded conditions. Unpaired student's two-tailed t-test was performed for each condition to determine differences in percent aggregation in the presence and absence of tau fibrils. P values are indicated along with summary information. * < 0.05, ** < 0.01, *** < 0.001 and **** < 0.0001.
